# Remote Physical Activity Monitoring in Neurological Disease: A Systematic Review

**DOI:** 10.1371/journal.pone.0154335

**Published:** 2016-04-28

**Authors:** Valerie A. J. Block, Erica Pitsch, Peggy Tahir, Bruce A. C. Cree, Diane D. Allen, Jeffrey M. Gelfand

**Affiliations:** 1 Graduate Program in Physical Therapy, University of California San Francisco/ San Francisco State University, San Francisco, California, United States of America; 2 Department of Physical Therapy and Rehabilitation Science, University of California San Francisco, San Francisco, California, United States of America; 3 University of California San Francisco Library, San Francisco, California, United States of America; 4 Multiple Sclerosis and Neuroinflammation Center, Department of Neurology, University of California San Francisco, San Francisco, California, United States of America; Xuanwu Hospital, Capital Medical Universty, CHINA

## Abstract

**Objective:**

To perform a systematic review of studies using remote physical activity monitoring in neurological diseases, highlighting advances and determining gaps.

**Methods:**

Studies were systematically identified in PubMed/MEDLINE, CINAHL and SCOPUS from January 2004 to December 2014 that monitored physical activity for ≥24 hours in adults with neurological diseases. Studies that measured only involuntary motor activity (tremor, seizures), energy expenditure or sleep were excluded. Feasibility, findings, and protocols were examined.

**Results:**

137 studies met inclusion criteria in multiple sclerosis (MS) (61 studies); stroke (41); Parkinson's Disease (PD) (20); dementia (11); traumatic brain injury (2) and ataxia (1). Physical activity levels measured by remote monitoring are consistently low in people with MS, stroke and dementia, and patterns of physical activity are altered in PD. In MS, decreased ambulatory activity assessed via remote monitoring is associated with greater disability and lower quality of life. In stroke, remote measures of upper limb function and ambulation are associated with functional recovery following rehabilitation and goal-directed interventions. In PD, remote monitoring may help to predict falls. In dementia, remote physical activity measures correlate with disease severity and can detect wandering.

**Conclusions:**

These studies show that remote physical activity monitoring is feasible in neurological diseases, including in people with moderate to severe neurological disability. Remote monitoring can be a psychometrically sound and responsive way to assess physical activity in neurological disease. Further research is needed to ensure these tools provide meaningful information in the context of specific neurological disorders and patterns of neurological disability.

## Introduction

Research over the last decade has examined accelerometer-based remote monitoring of physical activity in health and disease.[1–6] Wearable physical activity monitors have also become increasingly commonplace as consumer products, primarily marketed for fitness. When considering whether remote physical activity monitoring can inform decision-making for use in clinical populations, questions about validity, reliability, feasibility and responsiveness arise.[7–10]

Physical activity is typically defined as voluntary bodily movement using skeletal muscle that requires energy beyond resting levels.[11] Measurement of physical activity is important because of established links between physical inactivity and various morbidities.[5, 12, 13] Neurological disease can also increase the risk of physical inactivity secondary to associated disability.[14–17] Physical activity monitoring using accelerometers, pedometers, and gyroscopes has gained traction in healthcare, wellness and medical research.[5, 18–20] Monitoring can focus on gait, upper or lower limb function or other patterns of body movement or behavior. Potential variables that can be used to measure physical activity include step count, activity count, activity bouts, active minutes and energy expenditure. Remote physical activity monitoring provides a convenient way of assessing movement outside of the clinic setting and may correlate with disease-specific predictors, outcomes, or interventions.

However, remote measurement of physical activity in people with neurological disease has the potential to be complicated by neurological impairments such as gait abnormalities, weakness, spasticity or tremor that could confound remote measurement in these populations. While disease-specific examination and validation of remote physical activity is needed, systematically reviewing the literature across neurological disorders may reveal lessons about feasibility, implementation and interpretation that apply across neurological indications.

This systematic review summarizes research on remote physical activity monitoring in neurological diseases, including multiple sclerosis (MS), stroke, Parkinson’s disease (PD), dementia, traumatic brain injury (TBI), ataxia, epilepsy and migraine. To focus primarily on physical activity outside of the immediate clinical setting, studies were included that monitored physical activity for at least 24 hours.

## Methods

### Data Sources

Original research studies were identified from the PubMed/MEDLINE, CINAHL and SCOPUS databases. Once relevant articles were identified, they were located individually in the Web of Science database and in Google Scholar to examine citing and cited-by articles. The search strategy used a combination of MeSH (Medical Subject Headings) terms and keywords. The search terms used alone and in combination were categorized according to PICO: Population: “multiple sclerosis,” “parkinson*,” “stroke,” “cerebrovascular accident,” “brain injury,” “ataxia,” “headache,” “migraine,” and “epilepsy”. Intervention/ indicator: “acceleromet*,” “activity monitor*,” “free living physical activity,” “pedometer,” “wearable sensor*”.

Comparator/ Control: Not using the device. Inclusion criteria did not require studies to be intervention trials. Outcome: physical activity (measured heterogeneously e.g. step or activity count, movement count, bouts of activity)

We also examined articles that reported physical activity monitoring in samples with “heart disease” or “diabetes” to identify if sub-populations of neurological conditions were evaluated. A medical librarian (P.T.) advised on search strategy, search terms, and methodology.

#### Study Selection

Studies were included if they 1) recorded human physical activity, defined as voluntary (skeletal) muscle movement during daily functioning requiring energy expenditure [3]; 2) monitored subjects for ≥24 hours; 3) used remote monitoring via devices that employ accelerometers, gyroscopes and/or pedometers to measure physical activity and capture data remotely for subsequent analysis; 4) enrolled adults 18 years of age or older with a diagnosis of MS, stroke, PD, dementia, TBI, epilepsy, migraine, headache or ataxia; 5) and were published between January 2004 and December 2014. Studies were excluded that recorded involuntary motor activity such as seizures or tremor; focused on movement during sleep or examined sleep as the primary outcome; extrapolated measures for average step counts from shorter monitoring periods; measured total daily energy expenditure (such as daily calorie consumption or diet interventions) without physical activity monitoring; or measured global positioning satellite (GPS) data exclusively rather than more direct measurement or corroboration of physical activity. We also excluded case reports and case studies.

Two authors (V.B., E.P.) searched independently. Titles and abstracts were screened for relevance and supplementary review. One author (V.B.) manually searched the reference sections of complete manuscripts for additional articles. Consensus for meeting the eligibility criteria was achieved by comparing search results (V.B., E.P.).

#### Data extraction and Analysis

Data were extracted (V.B.) and checked (E.P., D.D.A., J.M.G), with final adjudication by consensus from two senior authors (D.D.A., J.M.G.). Variables included population studied; disease-specific severity levels; device name, placement and intent (i.e. patient behavior change or healthcare monitoring); intervention (if any); setting; demographic data; and study details, including design, funding sources and motivational factors (i.e. subject imbursement, visual display of data). Studies were graded for risk of bias based on methodology proposed by the Cochrane Collaborations [21] (see S1a and S1e Table). Conclusions and lessons learned across studies were summarized.

## Results

**Fig 1:**
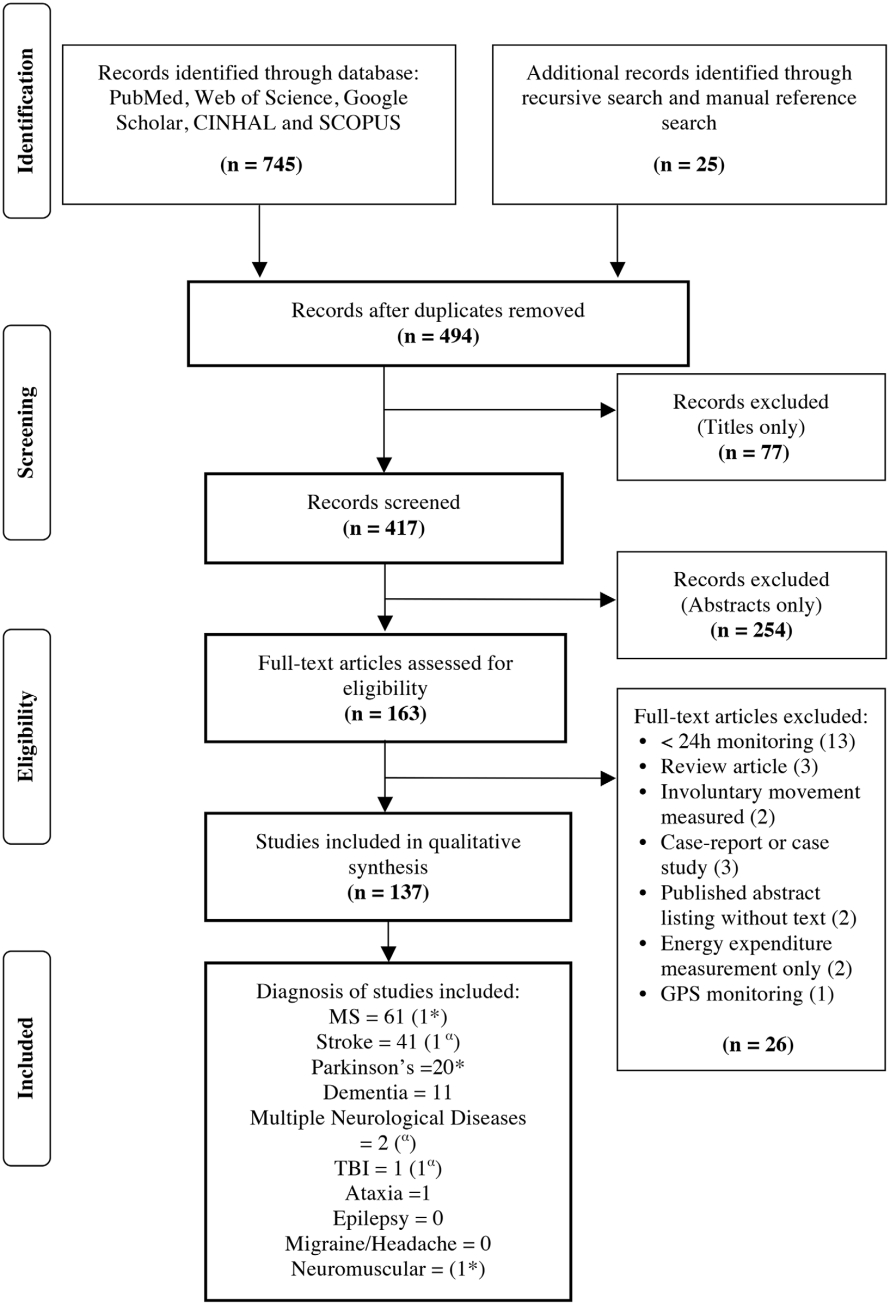
PRISMA Flow Diagram. Notes: * 1 Article includes multiple groups of neurological diagnosis—MS, Parkinson’s and neuromuscular disease—(Busse et al, 2004) ^α^ 1 Article includes TBI and Stroke (Fulk et al, 2014)

The systematic review identified 745 studies through the databases and an additional 25 articles through recursive and manual reference searches. Once eligibility criteria were applied, 137 studies remained (Fig 1 and S1 Fig) [22]. Individual studies are summarized in Tables 1–5. Table 6 (sections a-e) documents the sample characteristics. The risk of bias with level of evidence for interventional studies is reported in S1 Table. A description of the most common devices used in the included studies appears in S2 Table.

**Table 1:** Characteristics of Published Studies Recording Physical Activity via Remote Monitoring for >34 hours in People with Multiple Sclerosis.

Author / Year	MS phenotype	Number of people with RRMS	EDSS score or equivalent PDDS	Device name (Manufacturer)	Modality of the device	Study Design	Mean Age	Experimental Group N	Control Group N	Monitoring Length	Funding source
**Balantrapu et al, 2014** [23]	Mix of RRMS and SP/PPMS	69 of entire sample	0–5.5	ActiGraph GT3X (b*)	Walking /gait/ LE physical activity	Cross-Sectional	>50	44	40	7 days	Not stated/ unfunded
**Cavanaugh et al, 2011** [24]	Undefined/ Diagnosis of MS	N/A	< = 4.5 or >4.5	SAM (b*)	Walking /gait/ LE physical activity	Cross-Sectional	>50	21	N/A	7 days	Private Foundation
**Dlugonski et al, 2011** [25]	RRMS	21	0–5.5	ActiGraph 7164, OMRON pocket pedometer (b*)	Walking /gait/ LE physical activity	Intervention	18–50	21	N/A	7 days	Not stated/ unfunded
**Dlugonski & Motl, 2012** [26]	RRMS	46	0–5.5	ActiGraph 7164 (b*)	Walking /gait/ LE physical activity	Cross-Sectional	18–50	46	N/A	7 days	Not stated/ unfunded
**Dlugonski et al, 2013** [27]	Mix of RRMS and SP/PPMS	575 (89.2%)	0–5.5	ActiGraph 7164, ActiGraph GT3X / Yamax SW-200 (b*)	Walking /gait/ LE physical activity	Cross-Sectional	18–50	645	N/A	7 days	Private Foundation
**Doerksen et al, 2007** [28]	Mix of RRMS and SP/PPMS	174	Not stated	Yamax SW-200 (b*)	Walking /gait/ LE physical activity	Cross-Sectional	18–50	196	N/A	7 days	Not stated/ unfunded
**Filipovic Grcic et al, 2013** [29]	RRMS	82	0–5.5	SAM (b*)	Walking /gait/ LE physical activity	Cross-Sectional	18–50	82	N/A	7 days	Private Foundation
**Filipovic Grcic et al, 2011** [30]	RRMS	49	0–5.5	SAM (b*)	Walking /gait/ LE physical activity	Interventional	18–50	49	N/A	7 days	Not stated/ unfunded
**Gijbels et al, 2010** [31]	Mix of RRMS and SP/PPMS	23	0–5.5	SAM (b*)	Walking /gait/ LE physical activity	Cross-Sectional	18–50	50	N/A	7 days	Private Foundation
**Gosney et al, 2007** [32]	Mix of RRMS and SP/PPMS	174	Not stated	ActiGraph 7164, Yamax SW-200 (b*)	Walking /gait/ LE physical activity	Cross-Sectional	18–50	196	N/A	7 days	Private Foundation
**Hale et al, 2008** (c*)[33]	Undefined	N/A	Not stated	The TriTrac RT3 (Stayhealthy Inc.)	Walking /gait/ LE physical activity	Longitudinal	>50	11	9	7 days—repeated once	Private Foundation
**Klaren et al, 2013** [34]	Mix of RRMS and SP/PPMS	711	0–5.5	ActiGraph 7164 (b*)	Walking /gait/ LE physical activity	Cross-sectional and longitudinal	18–50	800	137	7 days	Private Foundation
**Klassen et al, 2008** [35]	Undefined/ Diagnosis of MS	N/A	0–5.5	The TriTrac RT3 (Stayhealthy Inc.)	Walking /gait/ LE physical activity	Cross-Sectional	18–50	30	9	2–6 days	Private Foundation
**Kos et al, 2007** [36]	Undefined/ Diagnosis of MS	N/A	0–5.5	ActiGraph 7164, ActiGraph GT3X (b*)	Walking /gait/ LE physical activity	Cross-Sectional	18–50	19	10	2–6 days	Private Foundation
**Lamers et al, 2013** [37]	SPMS	N/A	> or = 7	Motionlogger^®^ Basic, Accelerometers	Upper extremity/ arm movement	Cross-Sectional	>50	30	30	7 days	Private Foundation
**Learmonth et al, 2013** [38]	Mix of RRMS and SP/PPMS	65	0–5.5	ActiGraph GT3X (b*)	Walking /gait/ LE physical activity	Cross-Sectional	18–50	82	N/A	7 days	Private Foundation
**Learmonth et al, 2013** [39]	Mix of RRMS and SP/PPMS	79	0–5.5	ActiGraph GT3X (b*)	Walking /gait/ LE physical activity	Cross-Sectional	>50	96	N/A	7 days	Private Foundation
**Morris et al, 2008** [40]	Mix of RRMS and SP/PPMS	151 (SR)	Not stated	ActiGraph accelerometer (b*)	Walking /gait/ LE physical activity	Cross-Sectional	>50	173	136	7 days	Government
**Motl et al, 2013a** [41]	RRMS	269	0–5.5	ActiGraph 7164 (b*)	Walking /gait/ LE physical activity	Longitudinal	18–50	269	N/A	7 days—x 6 separated by 6 months	Private Foundation
**Motl et al, 2007b** [42]	Mix of RRMS and SP/PPMS	86(a*)	Not stated	ActiGraph 7164 (b*)	Walking /gait/ LE physical activity	Cross-Sectional	>50	133	N/A	7 days	Not stated/ unfunded
**Motl et al, 2010a** [43]	RRMS	26	0–5.5	ActiGraph 7164 (b*)	Walking /gait/ LE physical activity	Cross-Sectional	18–50	26	N/A	7 days	Not stated/ unfunded
**Motl & Dlugonski, 2011** [44]	RRMS	18	0–5.5	ActiGraph 7164, Digi-Walker SW-201 (b*)	Walking /gait/ LE physical activity	Interventional	18–50	18	N/A	7 days—repeated once	Not stated/ unfunded
**Motl et al, 2011a** [45]	Mix of RRMS and SP/PPMS	502	0–5.5	ActiGraph 7164 (b*)	Walking /gait/ LE physical activity	Cross-Sectional	18–50	561	N/A	7 days	Private Foundation / Government
**Motl et al, 2011b** [46]	Undefined/ Diagnosis of MS	N/A	6	SAM	Walking /gait/ LE physical activity	Cross-Sectional	>50	33	N/A	7 days	Private Foundation
**Motl et al, 2014a** [47]	Mix of RRMS and SP/PPMS	519	0–5.5	ActiGraph 7164 (b*)	Walking /gait/ LE physical activity	Longitudinal	18–50	536	N/A	7 days—repeated once	Private Foundation / Government
**Motl et al, 2014b** [48]	Mix of RRMS and SP/PPMS	67	Not stated	ActiGraph GT3X (b*)	Walking /gait/ LE physical activity	Longitudinal	18–50	82	N/A	7 days—repeated once	Private Foundation
**Motl et al, 2012a** [49]	Mix of RRMS and SP/PPMS	36	Not stated	ActiGraph 7164, Yamax SW-200 (b*)	Walking /gait/ LE physical activity	Cross-sectional	18–50	18	20	7 days—repeated once	Not stated/ unfunded
**Motl et al, 2009a** [50]	Mix of RRMS and SP/PPMS	246	0–5.5	ActiGraph 7164 (b*)	Walking /gait/ LE physical activity	Cross-Sectional	18–50	292	N/A	7 days	Government
**Motl et al, 2006a** [51]	Mix of RRMS and SP/PPMS	26	0–5.5	ActiGraph 7164, Yamax SW-200 (b*)	Walking /gait/ LE physical activity	Cross-Sectional	18–50	30	N/A	7 days—repeated once	Private Foundation
**Motl et al, 2007c** [52]	Mix of RRMS and SP/PPMS	174	Not stated	ActiGraph 7164, Yamax SW-200 (b*)	Walking /gait/ LE physical activity	Cross-Sectional	18–50	196	N/A	7 days	Not stated/ unfunded
**Motl et al, 2008a** [53]	Mix of RRMS and SP/PPMS	246	Not stated	ActiGraph 7164 (b*)	Walking /gait/ LE physical activity	Cross-Sectional	18–50	292	N/A	7 days	Government
**Motl et al, 2010b** [54]	RRMS	269	0–5.5	ActiGraph 7164 (b*)	Walking /gait/ LE physical activity	Cross-Sectional	18–50	269	N/A	7 days—repeated once	Government
**Motl & McAuley, 2009a** [55]	Mix of RRMS and SP/PPMS	239	0–5.5	ActiGraph 7164 (b*)	Walking /gait/ LE physical activity	Longitudinal	18–50	292	N/A	7 days—repeated once	Government
**Motl & McAuley, 2011** [56]	Mix of RRMS and SP/PPMS	246	0–5.5	ActiGraph 7164 (b*)	Walking /gait/ LE physical activity	Longitudinal	18–50	276	N/A	7 days—repeated once	Private Foundation
**Motl & McAuley, 2009b** [57]	Mix of RRMS and SP/PPMS	246	0–5.5	ActiGraph 7164 (b*)	Walking /gait/ LE physical activity	Longitudinal	18–50	276	N/A	7 days—repeated once	Government
**Motl & McAuley, 2009c** [58]	Mix of RRMS and SP/PPMS	246	0–5.5	ActiGraph 7164 (b*)	Walking /gait/ LE physical activity	Longitudinal	18–50	276	N/A	7 days—repeated once	Private Foundation
**Motl et al, 2013b** [59]	Mix of RRMS and SP/PPMS	215	0–5.5	ActiGraph GT3X (b*) Measuring wheel / GAITRite (CIR Systems, Inc.)	Walking /gait/ LE physical activity	Cross-Sectional	18–50	256	N/A	7 days	Not stated/ unfunded
**Motl et al, 2013c** [60]	Mix of RRMS and SP/PPMS	710	0–5.5	ActiGraph 7164, ActiGraph GT3X, Yamax SW-200 (b*)	Walking /gait/ LE physical activity	Observational	18–50	786	157	7 days	Private Foundation
**Motl et al, 2012b** [61]	Mix of RRMS and SP/PPMS	40	0–5.5	ActiGraph 7164 (b*), open-circuit spirometry system (TrueOne, Parvo Medics), GAITRite (CIR Systems, Inc.)	Walking /gait/ LE physical activity	Cross-Sectional	18–50	44	N/A	7 days	Private Foundation
**Motl et al, 2009b** [62]	Mix of RRMS and SP/PPMS	82	0–5.5	ActiGraph 7164 (b*)	Walking /gait/ LE physical activity	Cross-Sectional	>50	133	N/A	7 days	Not stated/ unfunded
**Motl et al, 2006c** [63]	Mix of RRMS and SP/PPMS	174	Not stated	ActiGraph 7164 (b*)	Walking /gait/ LE physical activity	Cross-Sectional	18–50	196	N/A	7 days	Not stated/ unfunded
**Motl et al, 2006b** [64]	Mix of RRMS and SP/PPMS	174	Not stated	ActiGraph 7164 (b*)	Walking /gait/ LE physical activity	Cross-Sectional	18–50	196	N/A	7 days	Not stated/ unfunded
**Motl et al, 2007d** [65]	Mix of RRMS and SP/PPMS	174	0–5.5	Yamax SW-200 (b*)	Walking /gait/ LE physical activity	Cross-Sectional	18–50	196	N/A	7 days	Not stated/ unfunded
**Motl et al, 2008b** [66]	Mix of RRMS and SP/PPMS	65	0–5.5	ActiGraph 7164 (b*)	Walking /gait/ LE physical activity	Cross-Sectional	18–50	80	N/A	7 days	Not stated/ unfunded
**Motl et al, 2007a** [67]	Mix of RRMS and SP/PPMS	171	Not stated	ActiGraph 7164, Yamax SW-200 (b*)	Walking /gait/ LE physical activity	Cross-Sectional	18–50	193	N/A	7 days	Not stated/ unfunded
**Pilutti et al, 2012** [68]	Mix of RRMS and SP/PPMS	134	0–5.5	ActiGraph GT3X (b*)	Walking /gait/ LE physical activity	Cross-Sectional	>50	168	N/A	7 days	Private Foundation
**Pilutti et al, 2014** [69]	Mix of RRMS and SP/PPMS	62	0–5.5	ActiGraph GT3X, Yamax SW-200 (b*)	Walking /gait/ LE physical activity	RCT	18–50	37	39	7 days—repeated once	Private Foundation
**Ranadive et al, 2012** [70]	Mix of RRMS and SP/PPMS	29	0–5.5	ActiGraph 7164 (b*)	Walking /gait/ LE physical activity	Cross-Sectional	18–50	33	33	7 days	Private Foundation
**Rietberg et al, 2014** [71]	Mix of RRMS and SP/PPMS	26	0–5.5	Vitaport, portable activity monitor (TEMEC Instruments)	Walking /gait/ LE physical activity	Cross-Sectional	18–50	43	26	1 day	Private Foundation
**Rietberg et al, 2010** [72]	Mix of RRMS and SP/PPMS	26	0–5.5	Vitaport, portable activity monitor (TEMEC Instruments)	Walking /gait/ LE physical activity	Cross-Sectional	18–50	43	N/A	2 days—x2 separated by 24hrs	Not stated/ unfunded
**Sandroff et al, 2013** [73]	Mix of RRMS and SP/PPMS	65	0–5.5	ActiGraph GT3X (b*)	Walking /gait/ LE physical activity	Longitudinal	18–50	82	N/A	7 days—repeated once	Private Foundation
**Sandroff et al, 2012** [74]	Mix of RRMS and SP/PPMS	66	0–5.5	ActiGraph 7164 (b*)	Walking /gait/ LE physical activity	Cross-Sectional	18–50	77	77	7 days	Private Foundation
**Sandroff & Motl, 2013** [75]	Mix of RRMS and SP/PPMS	37	0–5.5	ActiGraph 7164, ActiGraph GT3X (b*)	Walking /gait/ LE physical activity	Cross-Sectional	18–50	41	41	2–6 days	Private Foundation
**Scott et al, 2011** [76]	“Primary & Progressive MS”	N/A	Not stated	ActiGraph GT1M (b*)	Walking /gait/ LE physical activity	Cross-Sectional	>50	15	14	7 days	Private Foundation
**Shammas et al, 2014** [77]	Mix of RRMS and SP/PPMS	8	0–5.5	Move II activity sensor (movisens GmbH)	Walking /gait/ LE physical activity	Longitudinal	18–50	11	N/A	10 days every 3 months for a year	Not stated/ unfunded
**Snook et al, 2009** [78]	Mix of RRMS and SP/PPMS	58	0–5.5	ActiGraph 7164 (b*)	Walking /gait/ LE physical activity	Cross-Sectional	18–50	74	N/A	7 days	Not stated/ unfunded
**Snook & Motl, 2008** [79]	Mix of RRMS and SP/PPMS	62	0–5.5	ActiGraph 7164 (b*)	Walking /gait/ LE physical activity	Cross-Sectional	18–50	80	N/A	7 days	Not stated/ unfunded
**Sosnoff et al, 2010** [80]	Mix of RRMS and SP/PPMS	56	6	ActiGraph 7164 (b*)	Walking /gait/ LE physical activity	Cross-Sectional	>50	70	N/A	7 days	Not stated/ unfunded
**Ward et al, 2013** [81]	RRMS	25	Not stated	ActiGraph 7164 (b*)	Walking /gait/ LE physical activity	Cross-Sectional	18–50	25	26	7 days	Private Foundation
**Weikert et al, 2010** [82]	RRMS	269	Not stated	ActiGraph 7164 (b*)	Walking /gait/ LE physical activity	Cross-Sectional	18–50	269	N/A	7 days	Private Foundation
**Weikert et al, 2012** [83]	Mix of RRMS and SP/PPMS	56	Not stated	ActiGraph 7164 (b*)	Walking /gait/ LE physical activity	Cross-Sectional	18–50	33	33	7 days	Private Foundation

**Abbreviations**: EDSS = Kurtzke expanded disability scale; PDDS = Patient determined disease steps (correlated to EDSS): PDDS 1 = mild MS disability, PDDS mean 2 or 3 = moderate disability; RRMS = relapsing remitting multiple sclerosis; SP = Secondary progressive; PPMS = Primary progressive multiple sclerosis; SR = self reported, LE = lower extremity; N/A = not applicable; SAM = StepWatch Activity Monitor

^(a^*^)^ = 29/86 participants (34%) used an assistive device during the ambulatory tests.

^(b^*^)^ = Yamax SW-200 is a pedometer. Manufacturer: Yamax-Digiwalker, HRM USA INC, ActiGraph 7164 and GT3X use accelerometers. Manufacturer: Manufacturing Technology Inc /Health One Technology, SAM uses an accelerometer and microprocessor. Manufacturers: Orthocare Innovations/ or Manufacturer: Modus health llc, OMRON pocket pedometer. Manufacturer: HJ-720ITC, OMRON Corporation

^(c^*^)^ = Study included multiple cohorts with different neurological diagnoses.

**Table 2:** Characteristics of Published Studies Recording Physical Activity via Remote Monitoring for ≥24 hours in People with Stroke.

Author / year	Type of Stroke	Time Since Stroke	Device name (Manufacturer)	Modality of the device:	Study Design	Mean Age	Experimental group N	Control group N	Monitoring Length	Funding source
**Alzahrani et al, 2012** [84]	Undefined	> 3 months	Intelligent Device for Energy Expenditure and Physical Activity (b*)	Walking /gait/ LE physical activity	Cross-sectional	>50	42	N/A	2–6 days	Government
**Alzahrani et al, 2011** [85]	Undefined	> 3 months	Intelligent Device for Energy Expenditure and Physical Activity (b*)	Walking /gait/ LE physical activity	Cross-sectional	>50	42	21	2–6 days	Government
**Alzahrani et al, 2009** [86]	Undefined	> 3 months	Intelligent Device for Energy Expenditure and Physical Activity (b*)	Walking /gait/ LE physical activity	Cross-sectional	>50	42	N/A	2–6 days	Private foundation
**Askim et al, 2013** [87]	Ischemic / Hemorrhagic	8–14 days	PAL2, with a tilt switch (Gorman Promed Pty ltd)	Walking /gait/ LE physical activity	Cross-sectional	>50	28	N/A	2–6 days	Private foundation
**Baert et al, 2012** [88]	Ischemic / Hemorrhagic	Undefined	Yamax SW-200 (b*), Polar RS-400 HR monitor (Polar Electro Oy^®^)	Walking /gait/ LE physical activity	Cross-sectional	>50	16	N/A	2–6 days	Private foundation
**Barak et al, 2014** [89]	Ischemic / Hemorrhagic	> 14 days—3months	StepWatch Activity Monitor (b*)	Walking /gait/ LE physical activity	Cross-sectional	>50	408	N/A	2–6 days	Government
**Bowden et al, 2008** [90]	Undefined	> 3 months	StepWatch Activity Monitor (b*), GAITRite (CIR Systems, Inc.)	Walking /gait/ LE physical activity	Cross-sectional	>50	59	N/A	2–6 days	Government / Private foundation
**Butler & Evenson, 2014** [15]	Undefined	> 3 months	ActiGraph 7164 (b*)	Walking /gait/ LE physical activity	Cross-sectional	>50	262	524	7 days	Government
**Danks et al, 2014** [91]	Undefined	> 3 months	StepWatch Activity Monitor (b*)	Walking /gait/ LE physical activity	Open- label	>50	16	N/A	2–6 days	None listed
**de Niet et al, 2007** [92]	Hemorrhagic	> 3 months	Stroke-ULAM (Upper Limb Activity Monitor) (Biometrics Ltd)	Upper extremity/ arm movement	Cross-sectional	>50	18	5	1 day	None listed
**Dobkin et al, 2011** [93]	Undefined	> 3 months	The Medical Daily Activity Wireless Network (3M Corporation)	Walking /gait/ LE physical activity	Cross-sectional	>50	12	5	1 day	Government
**Frazer et al, 2013** [94]	Undefined	> 3 months	DynaPort MiniMod (McRoberts. B.V.)	Walking /gait/ LE physical activity	Cross-sectional	>50	14	N/A	7 days	None listed
**Fulk et al, 2010** [95]	Undefined	> 3 months	StepWatch Activity Monitor (b*)	Walking /gait/ LE physical activity	Cross-sectional	>50	12	N/A	7 days	None listed
**Fulk et al, 2014** (a*)[96]	Mixed population: TBI and Stroke (Undefined)	> 3 months	StepWatch Activity Monitor/ Fitbit Ultra/ Nike Fuelband/ Yamax SW-701 (b*)	Walking /gait/ LE physical activity	Cross-sectional	> 50	50	N/A	1 day	Not stated
**Gebruers et al, 2014** [97]	Ischemic / Hemorrhagic	≤ 7 days (acute)	Octagonal basic motion loggers (b*)	Upper extremity/ arm movement	Cross-sectional	>50	129	N/A	2–6 days	Government
**Gebruers et al, 2013** [98]	Ischemic / Hemorrhagic	≤ 7 days (acute)	Octagonal basic motion loggers (b*)	Upper extremity/ arm movement	Cross-sectional	>50	129	19	2–6 days	Private foundation
**Gebruers et al, 2008** [99]	Ischemic	≤ 7 days (acute)	Octagonal basic motion loggers (b*)	Both Upper extremity and Walking	Cross-sectional	>50	39	N/A	2–6 days	Private foundation
**Haeuber et al, 2004** [100]	Ischemic	> 3 months	StepWatch Activity Monitor (b*)	Walking /gait/ LE physical activity	Cross-sectional	>50	17	N/A	2–6 days	Government
**Knarr et al, 2013** [101]	Undefined	> 3 months	StepWatch Activity Monitor (b*)	Walking /gait/ LE physical activity	Cross-sectional	>50	98	N/A	2–6 days	Government
**Lang et al, 2007** [102]	Ischemic / Hemorrhagic	8–14 days	ActiGraph 7164 (MTI Health Services) (b*)	Upper extremity/ arm movement	Cross-sectional	>50	34	10	1 day	Government
**Lemmens et al, 2014** [103]	Undefined	> 3 months	Actiwatch-AW7 (CamNtech Ltd), Haptic Master (MOOG, Nieuw-Vennep, NL)	Upper extremity/ arm movement	RCT	>50	8	8	2–6 days	Private foundation
**Manns & Baldwin, 2009** [104]	Undefined	> 14 days—3months	StepWatch Activity Monitor (b*)	Walking /gait/ LE physical activity	Cross-sectional	>50	10	N/A	2–6 days	Private foundation
**Michael et al, 2009** [105]	Ischemic/ Hemorrhagic	> 3 months	StepWatch Activity Monitor (b*)	Walking /gait/ LE physical activity	Intervention	>50	10	N/A	5 days	Government / Private foundation
**Michielsen et al, 2012** [106]	Undefined	> 3 months	Stroke-Upper Limb-Activity Monitor (ULAM) (Biometrics Ltd)	Upper extremity/ arm movement	Cross-sectional	>50	38	18	1 day	None listed
**Moore et al, 2010** [107]	Unilateral supratentorial stroke	> 3 months	StepWatch Cyma Inc.	Walking /gait/ LE physical activity	Intervention	>50	20	N/A	1 m (5 days pre-post interventi-on)	Government
**Mudge et al, 2009** [108]	Undefined	> 3 months	StepWatch Activity Monitor (b*)	Walking /gait/ LE physical activity	RCT	>50	31	27	3 days x4	Government/ Private foundation
**Mudge & Stott, 2009** [109]	Undefined	> 3 months	StepWatch Activity Monitor (b*)	Walking /gait/ LE physical activity	Cross-sectional	>50	49	N/A	2–6 days	Government / Private foundation
**Mudge & Stott, 2008** [110]	Undefined	> 3 months	StepWatch Activity Monitor (b*)	Walking /gait/ LE physical activity	Cross-sectional	>50	40	N/A	2–6 days	None listed
**Rand & Eng, 2012** [111]	Ischemic / Hemorrhagic	> 14 days—3months	Actical (Philips Respironics)	Both Upper extremity and Walking	Cross-sectional	>50	60	40	2–6 days	Private foundation
**Rand et al, 2010** [112]	Undefined	> 3 months	Actical (Philips Respironics)	Walking /gait/ LE physical activity	Cross-sectional	>50	40	N/A	2–6 days	Private foundation
**Rand et al, 2009** [113]	Ischemic / Hemorrhagic	> 3 months	Actical (Philips Respironics)	Walking /gait/ LE physical activity	Cross-sectional	>50	40	N/A	2–6 days	Government
**Reiterer et al, 2008** [114]	Ischemic / Hemorrhagic	≤ 7 days (acute)	Actiwatch (b*)	Upper extremity/ arm movement	Longitudinal	>50	28	N/A	24 hrs x4, over 6 m	None listed
**Robinson et al, 2011** [115]	Undefined	> 3 months	VKRFitness Twin Step Pedometer (VKRFitness)	Walking /gait/ LE physical activity	Cross-sectional	>50	50	N/A	7 days	None listed
**Roos et al, 2012** [116]	Undefined	> 3 months	StepWatch Activity Monitor (b*)	Walking /gait/ LE physical activity	Cross-sectional	>50	51	14	2–6 days	Government
**Seitz et al, 2011** [117]	Ischemic (MCA)	≤ 7 days (acute)	Actiwatch (b*)	Upper extremity/ arm movement	Cross-sectional	>50	25	7	2–6 days	Government
**Shim et al, 2014** [118]	Undefined	> 3 months	Accelerometer (FITMETER 2010; KOREA) (U/D)	Upper extremity/ arm movement	Cross-sectional	>50	40	N/A	2–6 days	Private foundation
**Strommen et al, 2014** [119]	Transient ischemic attack / Ischemic	≤ 7 days (acute)	Actical (Philips Respironics)	Both Upper extremity and Walking	Cross-sectional	>50	100	N/A	2–6 days	Private foundation
**Thrane et al, 2011** [120]	Hemorrhagic	8–14 days	ActiGraph GT1M (b*)	Upper extremity/ arm movement	Cross-sectional	>50	31	N/A	1 day	None listed
**Uswatte et al, 2005** [121]	Undefined	> 3 months	Model 71256 Activity monitors (Manufacturing Technologies Inc.)	Upper extremity/ arm movement	Intervention	>50	10	10	2–6 days	Government
**Uswatte et al, 2006** [122]	Ischemic / Hemorrhagic	> 3 months	Wireless accelerometer (Manufacturing Technologies Inc.)	Upper extremity/ arm movement	Intervention	>50	82	87	2–6 days	Government
**Uswatte et al, 2009** [123]	Undefined	> 3 months	Wireless accelerometer (Manufacturing Technologies Inc.)	Upper extremity/ arm movement	Cross-sectional	>50	9	N/A	2–6 days	Government/ Private foundation
**Van der Pas et al, 2011** [124]	Undefined	> 3 months	ActiWatch AW7a (b*)	Upper extremity/ arm movement	Cross-sectional	>50	45	N/A	2–6 days	Private foundation

**Abbreviations**: N/A = not applicable, U/D = undefined, non-commercial, HR = heart rate, m = months

^(a^*^)^ = Study included multiple cohorts with different neurological diagnoses.

^(b^*^)^ = Intelligent Device for Energy Expenditure and Physical Activity. Manufacturers: MiniSun Company, Octagonal basic motion loggers. Manufacturers: Amubulatory Monitoring Inc., Yamax SW-200 is a pedometer. Manufacturer: Yamax-Digiwalker, HRM USA INC, ActiGraph 7164 and GT3X use accelerometers. Manufacturer: Manufacturing Technology Inc./ Health One Technology, StepWatch activity monitor use accelerometers and microprocessors. Manufacturer: Modus health llc/ or Manufacturers: Orthocare Innovations, Actiwatch. Manufacturer: Cambridge Neurotechnology, Fitbit uses accelerometers. Manufacturer: Fitbit Inc., Nike Fuel band uses accelerometers. Manufacturer: Nike Inc.

**Table 3:** Characteristics of Published Studies Recording Physical Activity via Remote Monitoring for ≥24 hours in People with Parkinson’s Disease.

Author / Year	Parkinson’s Level of Severity	Device name (Manufacturer)	Modality of the device	Study Design	Mean Age	Experimental group N	Control group N	Monitoring Length	Funding source
**Cancela et al, 2014** [125]	Mild	PERFORM (a*)	Walking /gait/ LE physical activity	Cross-sectional	> 50	11	N/A	2–6 days	Government/ Private foundation
**Cavanaugh et al, 2012** [126]	Mild /moderate	StepWatch activity monitor (b*)	Walking /gait/ LE physical activity	Longitudinal	>50	33	N/A	7 days—repeated once	Private foundation
**Chastin et al, 2010** [127]	Mild /moderate	ActivPAL (PAL Technologies Ltd)	Walking /gait/ LE physical activity	Cross-sectional	> 50	17	17	7 days	Device manufacturer (Involved—without monetary exchange)
**Dontje et al, 2013** [128]	Mild	TracmorD (Philips New Wellness Solutions, Lifestyle Incubator)	Walking /gait/ LE physical activity	Cross-sectional	> 50	467	N/A	7 days	Government /Private foundation
**El-Gohary et al, 2013** [129]	Mild /moderate	Opal sensors (APDM, Inc.)	Walking /gait/ LE physical activity	Cross-sectional	> 50	12	18	7 days	Government
**Ellis et al, 2011** [130]	Moderate /severe	StepWatch activity monitor (b*)	Walking /gait/ LE physical activity	Cross-sectional	> 50	164	96	7 days	Government /Private foundation
**Ford et al, 2010** [131]	Mild /moderate	StepWatch activity monitor (b*)	Walking /gait/ LE physical activity	Cross-sectional	> 51	12	N/A	7 days	Private foundation
**Garcia Ruiz & Sanchez Bernardos, 2008** [132]	Mild /moderate	AAM ActiTracÂ (ActiTracÂ 8.29 IM Systems)	Both Upper extremity and Walking	Cross-sectional	> 50	28	N/A	2–6 days	Not reported
**Hideyuki & Hitoshi, 2011** [133]	Mild /moderate	MVP-A3-05A-SD (MicroStone Corporation), Activity Monitoring And Evaluation System (Solid Brains Co., Ltd)	Walking /gait/ LE physical activity	Cross-sectional	> 50	9	N/A	1 day	Not reported
**Hideyuki & Hitoshi, 2014** [134]	Mild /moderate	MVP-A3-05A-SD (MicroStone Corporation)	Walking /gait/ LE physical activity	Interventional	>50	10	N/A	2–6 days	Private foundation
**Iluz et al, 2014** [135]	Mild /moderate /severe	DynaPort Hybrid, (McRoberts)	Walking /gait/ LE physical activity	Cross-sectional	> 50	33	N/A	2–6 days	Government
**Lord et al, 2013** [136]	Mild /moderate	ActivPAL (PAL Technologies Ltd)	Walking /gait/ LE physical activity	Cross-sectional	> 50	89	97	7 days	Government
**Moore et al, 2011** [137]	N/A	InvenSense IDG-300 (Freescale Semiconductor MMA7260QT)	Walking /gait/ LE physical activity	Cross-sectional	> 50	4	9	1 day	Government /Private foundation
**Pan et al, 2007** [138]	Mild /severe	ECOLOG (Ruputer Pro, Seiko Instruments)	Walking /gait/ LE physical activity	Cross-sectional	> 50	19	6	2–6 days	Government /Private foundation
**Rochester et al, 2006** [139]	Mild /moderate	Vitaport Activity Monitor (TEMEC Instruments Inc.)	Walking /gait/ LE physical activity	Cross-sectional	> 50	15	10	1 day	Private foundation
**Wallen et al, 2014a** [140]	Mild /moderate	ActiGraph GT3X, Yamax LS2000 (b*)	Walking /gait/ LE physical activity	Cross-sectional	> 50	51	61	7 days	Private foundation
**Wallen et al, 2014b** [141]	Mild /moderate	ActiGraph GT3X (b*)	Walking /gait/ LE physical activity	Cross-sectional	> 50	65	15	7 days	Government
**Weiss et al, 2014** [142]	Mild /moderate	DynaPort Hybrid system (McRoberts)	Walking /gait/ LE physical activity	Cross-sectional	> 50	107	N/A	2–6 days	Private foundation
**White et al, 2007** [143]	Mild /moderate	2 uni-axial (M92962) and 1 bi-axial (M92961) piezo-resistive accelerometers	Walking /gait/ LE physical activity	Longitudinal	> 50	9	N/A	24hrs x2, 48 hrs x1 (each separated by 1 week)	Government
**Yoneyama et al, 2013** [144]	Moderate/Severe	Mimamori-gait system (Mitsubishi Chemical)	Walking /gait/ LE physical activity	Cross-sectional	> 50	10	17	1 day	Not reported
**Busse et al, 2004** (c*)[145]	N/A	StepWatch activity monitor (b*)	Walking /gait/ LE physical activity	Longitudinal	> 50	10	10	7 days—repeated once	Government

**Abbreviations**: N/A = not applicable, ECOLOG = ECOlogical neurobehavior LOGger

^(a^*^)^ = PERFORM (Multi-parametric system for continuous effective assessment and Monitoring of motor status in Parkinson's disease and other neurodegenerative disease)

^(b^*^)^ = Yamax SW-200 is a pedometer. Manufacturer: Yamax-Digiwalker, HRM USA INC, ActiGraph 7164 and GT3X use accelerometers. Manufacturer: Manufacturing Technology Inc/ Health One Technology, StepWatch activity monitor use accelerometers and microprocessors. Manufacturer: Modus health llc/ or Manufacturers: Orthocare Innovations

^(c^*^)^ = Study included multiple cohorts with different neurological diagnoses.

**Table 4:** Characteristics of Published Studies Recording Physical Activity via Remote Monitoring for ≥24 hours in People with Dementia.

Author / Year	Presumed pathology	Cognitive score	Device name (Manufacturer)	Modality of the device	Study Design	Mean Age	Experimental group N	Control group N	Monitoring Length	Funding source
**David et al, 2012** [146]	AD	Mild	MicroMini (MotionLogger, Ambulatory- Monitoring)	Walking /gait/ LE physical activity	Cross-sectional	> 50	107	N/A	7 days	Government / Private foundation
**Erickson et al, 2013** [147]	AD /other dementia	AD/MCI/Control (U/D)	BodyMedia (SenseWear)	Walking /gait/ LE physical activity	Cross-sectional	> 50	39	28	2–6 days	Private foundation
**Gietzelt et al, 2014** [148]	AD	Moderate/ severe	Shimmer sensor (a*)	Walking /gait/ LE physical activity	Longitudinal	> 50	40	N/A	7 days—x4	Private foundation
**Gietzelt et al, 2013** [149]	Dementia diagnosis	MMSE cut off <24/30: Mild/ Moderate	Shimmer sensor (a*)	Walking /gait/ LE physical activity	Cross-sectional	> 50	10	10	7 days	Not stated
**Greiner et al, 2007** [150]	AD	Moderate. Mean MMSE 11.2 ± 5.5.	Activity monitoring system (Matrix Co.)	Walking /gait/ LE physical activity	Cross-sectional	> 50	12	N/A	7 days	Private foundation
**Hoffmeyer et al, 2012** [151]	AD	Moderate	Shimmer sensor (U/D)	Walking /gait/ LE physical activity	Cross-sectional	> 50	16	16	2–6 days	Not stated
**James et al, 2012** [152]	Dementia diagnosis	Mild / moderate	Actical^®^ (Mini Mitter)	Walking /gait/ LE physical activity	Cross-sectional	> 50	70	624	Median 9 (range 2–16) days	Government / Private foundation
**Kirste et al, 2014** [153]	AD	Mild	Shimmer sensors (U/D)	Walking /gait/ LE physical activity	Cross-sectional	> 50	23	23	2–6 days	Not stated
**Nagels et al, 2007** [154]	AD / Lewy body/ Frontotempo-ral /other dementia	Moderate	Octagonal basic motionlogger (Ambulatory monitoring)	Both Upper extremity and Walking	Cross-sectional	> 50	110	N/A	2–6 days	Private foundation
**Nagels et al, 2006** [155]	AD / Lewy body/ Frontotempo-ral /other dementia	Moderate/ severe	Octagonal basic motionlogger (Ambulatory monitoring)	Both Upper extremity and Walking	Cross-sectional	> 50	110	N/A	2–6 days	Private foundation
**Yuki et al, 2012** [156]	Frontotempo-ral / other dementia	Mild	Lifecorder (Suzuken)	Walking /gait/ LE physical activity	Longitudinal	> 50	774	N/A	2–6 days	Private foundation

**Abbreviations**: MCI = mild cognitive impairment, AD = Alzheimer’s Disease, U/D = undefined, MMSE = mini mental state exam, N/A = not applicable, LE = Lower extremity, N = number

(a*) = Shimmer—Wireless Sensor Platform for Wearable Applications. Internet: http://www.shimmer-research.com

**Table 5:** Characteristics of Published Studies Recording Physical Activity via Remote Monitoring for ≥24 hours in People with Traumatic Brain Injury, Ataxia and Studies with Multiple Conditions.

Author / Year	Pathology/ Diagnosis	Time Since Diagnosis/Injury	Device name (Manufacturer)	Modality of the device	Study Design	Mean Age	Experimental group N	Control group N	Monitoring Length	Funding source
Fulk et al, 2014**[96]	TBI / Stroke	> 3 months	StepWatch Activity Monitor/ Fitbit Ultra/ Nike Fuelband/ Yamax DigiWalker SW-701 (a*)	Walking /gait/ LE physical activity	Cross-sectional	> 50	50	N/A	1 day	Not stated
**Hassett et al, 2014** [157]	TBI	> 3 months	ActiGraph GT3X (a*)	Walking /gait/ LE physical activity	Cross-sectional	18–50	30	N/A	7 days	Private foundation
**Subramony et al, 2012** [158]	Spino-cerebellar Ataxia	>5–10 years	StepWatch Activity Monitor (a*)	Walking /gait/ LE physical activity	Cross-sectional	> 50	19	N/A	7days	Not stated
**Hale et al, 2008** (b*)[33]	Stroke/PD/ MS	> 6 months stroke (N/A others diagnosis)	The TriTrac RT3 (Stayhealthy Inc.)	Walking /gait/ LE physical activity	Cross-sectional	> 50	38	9	Av. of 3 days and 7 days, repeated once	Private Foundation
**Busse et al, 2004** (b*)[145]	PD, MS, Neuromuscular	N/A	StepWatch activity monitor (a*)	Walking /gait/ LE physical activity	Cross-sectional	> 50	10	10	7 days repeated once	Government

**Abbreviations**: TBI = Traumatic brain injury, PD = Parkinson’s disease, MS = Multiple sclerosis, N/A = not applicable, LE = lower extremity, Av. average

^(a^*^)^ = Yamax DigiWalker SW-701 is a pedometer. Manufacturer: YAMAX Health & Sports Inc, ActiGraph GT3X use accelerometers. Manufacturer: Manufacturing Technology Inc /Health One Technology, StepWatch activity monitor uses an accelerometer and microprocessor. Manufacturers: Orthocare Innovations/ or Manufacturer: Modus health llc, Fitbit uses an accelerometer. Manufacturer: Fitbit Inc., Nike Fuelband uses accelerometers. Manufacturer: Nike Inc.

^(b^*^)^ = Study included multiple cohorts with different neurological diagnoses

** This study is included in Table 1b—Stroke.

**Table 6:** Summary Characteristics of Studies by Neurological Diagnosis.

**Section a**:			**MULTIPLE SCLEROSIS**
	**Number of studies**	**Percent (%)**	**Notes**
***Number of Articles Identified***	***61***		*Median year published*: *2011*
**Mean Age of Participants / Years**			
(18–50)	49	80.3	
(>50)	12	19.7	
**Sex**			Greater % of females
Both	59	96.7	
Female only	2	3.3	
**MS Phenotype**			- 79% of participants in all included MS studies had RRMS
(RRMS)	10	16.4	
(SPMS)	1	1.6	
(Relapsing and Progressive)	44	72.2	
(“Diagnosed with MS” / Undefined)	6	9.8	
**Disability Level (EDSS and PDDS equivalent)**			EDSS/PDDS: 6 (2, 3.3%), ≥7 (2, 3.3%)
(0–5.5)	40	65.6	
(>5.5)	4	6.7	
(Not stated)	17	27.9	
**Mean Disease Duration / Years**			0–1 year (0.0%), >1 year– 5 years (0.0%) when reported
(>5–10)	28	45.9	
(>10–20)	29	47.5	
(>20)	1	1.6	
(Not stated)	3	4.9	
**Reporting of Paralysis/Paresis**	0	0.0	
**Reporting of Tremor**	1	1.6	- As an exclusion criteria [36]
**Monitoring Length**			
(1 day)	1	1.6	
(2–6 days)	3	4.9	
(7 days)	41	67.2	
(7 days, repeated once)	13	21.3	
(2 days, x2—separated by 24 hours)	1	1.6	
(7 days, every 6 months—for 2.5yrs)	1	1.6	
(10 days, every 3 months—for 1yr)	1	1.6	
**Device Used in Physical Activity Monitoring**			
*(ActiGraph 7164)	38	62.3	
*(ActiGraph GT3X)	12	19.7	
*(Yamax SW-200 pedometer)	10	16.4	
(Other)	10	16.4	
(StepWatch Activity Monitor)	5	8.2	
(RT3 accelerometer)	1	1.6	
**Device Intent**			
(Healthcare monitoring)	53	86.9	
(Patient behavior change)	6	9.8	
(Both)	2	3.3	
**Device Placement**			
(Unaffected hip)	43	70.5	
(Posterior waist)	4	6.6	
(Unaffected ankle)	4	6.6	
(Not stated)	4	6.6	
(Right hip)	3	4.9	
(Both wrists)	2	3.3	
(Right ankle)	1	1.6	
**Device Modality**			- Both (0, 0.0%)
(Walking/ gait activity)	60		
(Upper extremity/arm activity)	1		
**Defined Acceptable Full Days Monitoring**			
(Yes)	44	74.6	**- For yes (44)**: > 10 hours of data (30, 68.2%), < 60 minutes of zero scores (24, 54.5%), >3 days per week (7, 15.9%), >5 days per week (2, 4.5%), undefined (10, 22.7%)
(No)	15	25.4	
**Study Setting**			Clinic (0, 0.0%)
(Home/ community)	48	78.7	
(Both Clinic and Home)	13	21.3	
**Study Design**			
(Observational)	57	93.4	
(Interventional)	4	6.6	
**Total N range**			
[Control and neurological group]			
(Lowest N)	11	-	
(Greatest N)	943	-	
**Neurological groups N range**			
(Lowest N)	11	-	
(Greatest N)	800	-	
**Study Funding**			Device manufacturer (0, 0.0%)
(Private Foundation)	31	50.8	
(Not stated/ unfunded at time of publication)	22	36.1	
(Government)	6	9.8	
(Both)	2	3.3	
**Section b**:			**STROKE**
***Number of Articles Identified***	***41***		*Median year published*: *2011*
**Mean Age of Participants**	>50	100	
**Sex**	Both	100	
**Type of Stroke**			
(Undefined)	23	56.1	
(Both Ischemic and Hemorrhagic)	11	26.8	Ischemic (Middle cerebral artery: 2, 14.3%, undefined: 13, 92.9%)
(Ischemic)	3	7.3	
(Hemorrhagic)	2	4.9	
(Transient Ischemic Attack)	1	2.4	
(other)	1	2.4	
**Time Since Stroke**			
(≤7 days—acute)	6	14.6	
(8–14 days)	3	7.3	
(>14 days– 3 months)	3	7.3	
(>3 months)	28	68.3	
(Undefined)	1	2.4	
**Reporting of Paralysis/Paresis**			
(Yes)	38	92.7	
(No)	3	7.3	
**Reporting of Tremor**			
(Yes)	1	2.4	
(No)	40	97.6	
**Monitoring Length**			
(1 day)	5	12.2	
(2–6 days)	28	68.3	
(5 days)	1	2.4	
(7 days)	4	9.8	
(24 hours at 4 time points over 6 months)	1	2.4	
(3 days at: baseline x2, post-Intervention and 3 month follow-up)	1	2.4	
(4 weeks: data from 5 days before and after intervention)	1	2.4	
**Device Used in Physical Activity Monitoring**			
(Other)	25	61.0	
(ActiGraph 7164)	2	4.9	
(StepWatch)	13	31.7	
(Intelligent Device for Energy Expenditure and Physical Activity)	3	7.3	
(Yamax SW-200 pedometer)	1	2.4	
**Device Intent**			
(Healthcare monitoring)	34	82.9	
(Behavior change)	3	7.3	
(Both)	4	9.8	
**Device Modality**			
(Walking/ gait activity)	24	58.5	
(Upper extremity/arm activity)	14	34.1	
(Both)	3	7.3	
**Defined Acceptable Full Day**			
(Yes)	27	65.9	
(No)	14	34.1	
**Study Setting**			
(Home)	18	43.9	
(Home and Out patient)	10	24.4	
(Home and Hospital—acute care)	1	2.4	
(Hospital—acute care)	9	22.0	
(Hospital—acute care and Out patient)	1	2.4	
(Out patient)	2	4.9	
**Study Design**			
(Observational)	34	82.9	
(Interventional)	7	17.1	
**Blinding**			
(Yes)	7	17.1	-If Yes: clinician and analyst (3/5), participant (3/5), researcher and analyst (1/5)
(No)	34	82.9	
**Total N range**		-	
(Lowest N)	10		
(Greatest N)	786		
**Neurological groups N range**		-	
(Lowest N)	8		
(Greatest N)	408		
**Study Funding**			
(Government)	14	34.1	
(Private Foundation)	12	29.3	
(Not stated/unfunded at time of publication)	10	24.4	
(Both)	5	12.2	
**Section c**:			**PARKINSON’S DISEASE**
***Number of Articles Identified***	***20***		*Median year published*: *2012*
**Mean Age of Participants**	>50	100	
**Sex**	Both	100	
**Reporting of Paralysis/Paresis**			
(No)	20	100	
**Reporting of Tremor**			
(Yes)	7	35.0	
(No)	13	65.0	
**Monitoring Length**			
(1 day)	4	20.0	
(2–6 days)	6	30.0	
(7 days)	8	40.0	
(7 days—repeated once)	1	5.0	
24 hrs x2, 48 hrs once (each separated by 1 week)	1	5.0	
**Device Used in Physical Activity Monitoring**			
(Other)	13	65.0	
(StepWatch)	3	15.0	
(ActiGraph GT3X)	2	10.0	
(ActivPAL)	2	10.0	
**Device Intent**			
(Healthcare monitoring)	19	95.0	
(Behavior change)	1	5.0	
**Device Placement**			
(Anterior waist)	5	25.0	
(Posterior waist)	3	15.0	
(Both ankles)	3	15.0	
(Both wrists)	3	15.0	
(Hip unaffected or non-dominant)	1	5.0	
(Multiple limbs)	5	25.0	
**Device Modality**			
(Walking/ gait activity)	19	95.0	
(Upper extremity/arm activity)	0	0.0	
(Both)	1	5.0	
**Defined Acceptable Full Day**			
(Yes)	12	60.0	**- For Yes**: greater than 10 hours minutes of zero scores (2), more than 3 days per week (3), undefined (5)
(No)	8	40.0	
**Study Setting**			-
(Home)	13	65.0	
(Home and Out patient)	6	30.0	
(Hospital—acute care)	1	5.0	
**Study Design**			
(Observational)	19	95.0	- Cross sectional (17), longitudinal (2)
(Interventional)	1	5.0	
**Blinding**			
(No)	18	90.0	
(Yes)	2	10.0	**- If Yes**, who was blinded: participants (1), analyst (1)
**Total N range**		-	
(Lowest N)	4		
(Greatest N)	467		
**Neurological groups N range**		-	
(Lowest N)	4		
(Greatest N)	467		
**Study Funding**			
(Government)	5	25.0	
(Private Foundation)	6	30.0	
(Both)	6	30.0	
(Not stated/ unfunded at time of publication)	3	15.0	
(Device manufacturer)	(0)	0.0	- 1 author: co-inventor of the device, not involved in data collection or analysis of results
**Section: d**			**DEMENTIA**
***Number of Articles Identified***	***11***		*Median year published*: *2012*
**Mean Age of Participants**	>50	100	
**Sex**			
(Both)	11	100	
**Cognitive Scoring**			
(Mild)	3	27.3	
(Moderate)	3	27.3	
(Mild—Moderate)	3	27.3	- MMSE cut off <24/30: Mild/ Moderate
(Moderate—Severe)	2	18.2	
**Presumed Pathology**			
(Alzheimer’s)	5	45.5	
(Probable Alzheimer’s and other dementia)	1	9.1	
(Alzheimer’s / Lewy body/ Frontotemporal /other dementia)	2	18.2	
(Dementia diagnosis)	2	18.2	
(Frontotemporal / other dementia)	1	9.1	
**Reporting of Paralysis/Paresis**			
(No)	11	100	
**Reporting of Tremor**			
(Yes)	1	9.1	
(No)	10	90.9	
**Monitoring Length**			
(2–6 days)	6	54.5	
(7 days)	3	27.3	
(7 days—repeated x4)	1	9.1	
(Median of 9 days)	1	9.1	
**Device Used in Physical Activity Monitoring**			
(Other)	11	100	
**Device Intent**			
(Healthcare monitoring)	11	100	
(Behavior change)	0	0.0	
**Device Placement**			
(Left or non-dominant wrist)	3	27.3	
(Right or dominant wrist	1	9.1	
(Both wrists)	1	9.1	
(Left ankle)	1	9.1	
(Both ankles)	1	9.1	
(Right hip)	1	9.1	
(Other/ Multiple limbs)	3	27.3	
**Device Modality**			
(Walking/ gait activity)	9	81.8	
(Upper extremity/arm activity)	0	0.0	
(Both)	2	18.2	
**Defined Acceptable Full Day**			
(Yes)	8	72.7	**-For Yes**: greater than 10 hours of data (2), less than 60 minutes of zero scores (1), more than 3 days per week (1), undefined (4)
(No)	3	27.3	
**Study Setting**			
(Home)	8	72.7	
(SNF)	3	27.3	
**Study Design**			
(Observational)	11	100	- Cross sectional (9), longitudinal (2)
**Blinding**		1	
(No)	11	00	
**Total N range**			
(Lowest N)	12		
(Greatest N)	774	-	
**Neurological groups N range**		-	
(Lowest N)	10		
(Greatest N)	774		
**Study Funding**			
(Private Foundation)	6	54.5	
(Both Government and Private)	2	18.2	
(Not stated/ unfunded at time of publication)	3	27.3	
**Section e**:			**TRAUMATIC BRAIN INJURY**
***Number of Articles Identified***	***1***		
**Mean Age of Participants**			
(18–50)	1	100	
(>50)	0	0.0	
**Sex**	Both	100	
**Time Since Diagnosis**			
(> 3 months)	1	100	
**Device Used in Physical Activity Monitoring**			
(ActiGraph GT3X)	1	100	
**Device Modality**			
(Walking/ gait activity)	1	100	
**Monitoring Length**			
(7 days)	1	20.0	
**Neurological groups N**	30	-	
**Funding**			
(Private Foundation)	1	100	
			**ATAXIA**
***Number of Articles Identified***	***1***		
**Mean Age of Participants**	0		
(18–50)	1		
(>50)			
**Sex**	Both	100	
**Device Modality**			
(Walking/ gait activity)	1	100	
**Monitoring Length**			
(7 days)	1	100	
**Neurological groups N**	19	-	
**Funding**			
(Not stated /unfunded at time of publication)	1	100	
			**ACROSS MULTIPLE NEUROLOGICAL DIAGNOSES**
***Number of Articles Identified***	***3***		
**Diagnosis**	1	33.3	
(TBI/ Stroke)			
(Stroke/PD/MS)	1	33.3	
(PD/AD/Neuromuscular disorder)	1	33.3	
**Mean Age of Participants**			
(18–50)	0	0.0	
(>50)	3	100	
**Sex**	Both	100	
**Device Modality**			
(Walking/ gait activity)	3	100	
**Monitoring Length**			
(1 day)	1	33.3	
(7 days)	1	33.3	
(Average of 3 days and 7 days, repeated once)	1	33.3	
**Neurological groups N range**			
(Lowest N)	10	-	
(Greatest N)	50	-	
**Funding**			
(Government)	1	33.3	
(Private Foundation)	1	33.3	
(Not stated /unfunded at time of publication)	1	33.3	

**Abbreviations**: yrs: years, hrs: hours, MMSE: Mini-Mental Status Examination, SNF: Skilled Nursing Facility, N: number,

*: Used in conjunction with another activity monitor TBI: Traumatic Brain Injury, MS: Multiple Sclerosis, PD: Parkinson’s disease, AD: Alzheimer’s disease.

### Multiple Sclerosis

The majority of the 61 studies (60/61, 98.4%) that remotely monitored activity in MS [23–82] (Tables 1 and 6 section a) measured physical activity by walking; one study focused on upper extremity movement.[83] The length of continuous monitoring ranged from 3 to 7 days for each discrete measurement period [33, 66] with 7 days being the measurement paradigm for the majority (41/61, 67.2%) of studies. Most of the studies (44/61, 72.2%) [23, 27, 28, 31, 32, 34, 37, 39, 41, 44, 46–56, 58–74, 76–79, 82, 84] included both relapsing and progressive MS phenotypes; >78% of participants had relapsing MS. Although MS disease duration varied, studies primarily included persons with disease duration of less than 20 years. Fifty-two studies focused on people having mild to moderate disability (able to walk without a cane or support) [23, 25–27, 29–31, 34–37, 40, 42–44, 47–50, 52, 55–61, 64, 65, 67–74, 76, 78, 79], and only two studies reported inclusion of people with greater levels of disability (requiring a walker or wheelchair for mobility).[24, 83] One research group (Department of Kinesiology and Community Health, University of Illinois at Urbana-Champaign, Urbana, Illinois) authored 49/61 studies (81.7%)[23, 25–28, 32, 34, 37, 39–69, 72–74, 78–82]; results from studies conducted by other groups generally corroborated this group’s results. No studies reported direct research funding by monitoring device manufacturers.

In two studies focused on people with MS, average daily activity and step count measured via wearable accelerometers correlated with performance-based and self-reported walking mobility and physical activity.[78, 81] A third study observed that accelerometers correlate only with performance-based measures of walking (6-minute walk; [6MW,] and the Timed-Up and Go, test; [TUG]) and not self-reported walking activity.[82]

People with MS record lower levels of physical activity than the general population and unaffected controls.[23, 27, 28, 32, 34, 37, 38, 52, 63, 66, 69, 73, 75, 77, 80] People with MS also frequently fail to reach daily levels of intensity and duration recommended for the general population.[85] Lower physical activity levels in MS are associated with higher levels of disability and lower scores in a range of clinical and self-reported outcomes such as walking speed and endurance (Timed 25-Foot Walk [23, 24, 29, 30, 37, 38, 62, 68, 76], 2-minute walk and 6MW [23–25, 31, 37, 38, 44, 49, 60, 67, 80, 82]), fatigue (i.e. Fatigue Severity Scale)[40, 48, 57], depression (i.e. Hospital Anxiety and Depression Scale [48, 57, 68]), self-efficacy, [39, 40, 62] and balance (Berg Balance Scale [24, 31, 38], TUG [23, 31, 80, 82]). Higher levels of physical activity correlate significantly with better performance on mobility measures in the clinic, self-reported disability questionnaires and cognitive processing speed.[41, 45, 53] Lower physical activity in MS correlates with age, [64] disease duration, [34] progressive forms of MS, [27] spasticity, [23] and unemployment, [27] but not race.[34] However, rate of disability accumulation over 6 months was similar in an active versus a more sedentary group in one study.[50] Of the 4 studies in MS that tested interventions, internet-based interventions appear to be beneficial in promoting objective and self-reported physical activity and are associated with decreased disability.[26, 30, 42, 68]

### Stroke

More than half of the studies (24/41 studies, 58.5%)[15, 86–125] that reported on activity monitoring post-stroke (Tables 2 and 6 section b) measured walking or gait; 14 studies (34.1%)[94, 98, 99, 103, 104, 109, 115, 118, 119, 121–125] assessed upper extremity or arm movement; and 3 (7.3%)[100, 112, 120] measured both arm movement and walking. One study included participants with either a diagnosis of stroke (n = 30) or TBI (n = 20). This study is listed under both diagnostic headings and results are analyzed by diagnosis group.[126] Monitoring duration was usually between 2 and 6 days (28 studies, 68.3%)[86–93, 98–102, 104, 105, 110–114, 117–120, 122–125], although one study monitored step count for 4 weeks, reporting change in daily average steps between the 5 days prior and post intervention.[106] Monitoring usually commenced between 3 and 6 months after the stroke (28, 68.3%)[15, 86–88, 92–97, 101, 102, 104, 106–111, 113, 114, 116, 117, 119, 122–125]. Fewer than 40% of studies reported details about the type of stroke (i.e. ischemic or hemorrhagic and/or neuroanatomical localization). The presence and side of paralysis or paresis was reported in 92.7% (38/41)[15, 86–101, 103, 104, 106–125] of the articles; one article reported on the presence or absence of tremor as a potential confounder.[125] During monitoring, participants were in the “home/ community” or “hospital—acute care” settings; none of these studies specifically monitored patient activity in acute rehabilitation or at skilled nursing facilities.

Post-stroke, people tend to have a lower frequency of moderate to vigorous bouts of physical activity and are less likely to reach generally recommended minimum levels of physical activity than healthy controls.[15, 86] However, one study found that the “time participants spent on their feet” was similar to healthy controls.[86] Lower physical activity level post-stroke is associated with poor balance and greater depression scores.[88] Four intervention studies were identified: 3 aimed at improving arm function, [104, 122, 123] and 1 successfully increased daily step counts using a goal-directed step activity-monitoring program.[93] An observational study showed little change in daily limb use with accelerometer results, despite significant improvements in clinical measures.[112] Measuring both upper extremities post-stroke facilitated differentiation of uni- vs. bi-manual tasks, distribution of arm usage, and comparison of impaired vs. unimpaired arm function.[104] Spontaneous early arm movement activity was associated with greater neurological recovery post stroke, [118] although results varied regarding prediction of upper extremity recovery. [100, 119–121, 124, 125]

### Parkinson’s Disease

All 20 studies [127–146] that reported on activity monitoring in PD (Tables 3 and 6 section c) measured physical activity through walking. Durations of monitoring were mostly for 2–6 days (6, 35.0%)[127, 134–136, 140, 144] or 7 days (8, 40.0%).[129–133, 137, 142, 143] Thirty-five per cent of studies (7/20) reported on the presence or absence of tremor as a potential confounder.[127, 134–136, 139–141]

One activity-monitoring device (DynaPort Hybrid) was able to differentiate between ON/OFF phases and detect “missteps/ near falls” in people with PD in the clinic and home environments.[136] Participants wore the device in the clinic while missteps were induced, an algorithm was developed to detect deviations from their gait patterns, and the algorithms were validated during an additional three days of device wear-time outside the clinic. Abnormal gait patterns, such as lower amplitude and greater step-to-step variability, were associated with fall risk in people with PD whereas total walking amount was not.[144]

People with PD tend to take fewer steps and do shorter bouts of physical activity than the general population.[130, 137, 147] A reduction in total number of steps per day correlates with PD progression, [128] and milder severity of PD is associated with higher physical activity levels.[135] People with PD tend to have a smaller number of longer sedentary periods than healthy controls, although total sedentary time is similar.[129] An intervention study aimed at increasing physical activity in people with PD resulted in increased muscle strength and flexibility, self-directed exercise frequency and duration, reduced fear of falls, but no overall change in the total amount of physical activity.[135]

### Dementia

Nine [148–156] of the 11 [148–158] studies (81.8%) that reported on activity monitoring in dementia (Tables 4 and 6 section d) measured physical activity as walking. Two studies focused on upper extremity or arm movement in addition to walking or gait.[157, 158] Monitoring typically lasted 2–6 days (6/11 studies, 54.5%).[149, 153, 155–158] Most studies involved people with a presumed Alzheimer's dementia or a combination of Alzheimer’s dementia and frontotemporal or Lewy Body dementias (8/11 studies, 72.7%).[148–150, 152, 153, 155, 157, 158] Severity of cognitive dysfunction was usually mild to moderate (9/11 studies, 81.8%).[148, 149, 151–157, 159] Only 2 studies involved people with severe cognitive disability.[150, 158]

Physical activity level in people with dementia depended on stage of disease. People with mild Alzheimer’s dementia have lower mean physical activity (associated with apathy and more daytime napping)[148] and lower step count per day [149] compared to people with mild cognitive impairment (MCI) or healthy controls. Monitoring was feasible in people with cognitive impairment [149, 155] and accelerometry was able to distinguish partners with and without early Alzheimer’s disease even before deficits were clinically visible.[155] Monitoring in people with dementia distinguished “intensive wandering behavior,” which, when assessed along with estimations of energy expenditure, facilitated accurate calculation of nutritional requirement.[152]

### Traumatic Brain Injury

The single study in TBI concluded that 7 days of accelerometry was feasible in 30 people more than 3 months post-TBI (adherence >86%). Physical activity was below recommended levels.[160] Data were more reliable than a self-reported physical activity questionnaire to determine amount, but not type of, moderate to vigorous physical activity.[160]

### Ataxia

In a single study of physical activity monitoring in ataxia, 19 participants with spinocerebellar ataxia wore a step activity monitor for 7 days; greater physical activity was associated with shorter disease duration and lower disability scores.[161]

The remaining studies that reported physical activity monitoring in mixed populations [33, 126, 147] measured walking activity or gait (Tables 5 and 6 section e). One study observed 50 people with either TBI or stroke over the age of 50 and greater than 3 months post injury assessing various activity monitoring systems.[126] Another study evaluated a tri-axial accelerometer (TriTrac RT3) over 7 days in a study sample of patients with stroke (> 6 months in duration) (20), PD (7), or MS (11), and sedentary healthy controls (9).[33] Mobility was more accurately assessed using 7-day activity monitoring than with a patient reported measure. A third study measured step count in participants with PD (10), MS (10), primary muscle disorder (10) and healthy controls (30) over 7 days in free-living conditions.[147] Neurological patients were observed to have a lower level of physical activity than healthy controls.

### Reliability and Validity

Many studies provided evidence of the reliability of various devices. For the StepWatch Activity Monitor post-stroke, the test-retest interclass correlation coefficient (ICC) values were 0.93–0.99 over a minimum of 3 days.[110] Other studies documented similar ICC values for Actical accelerometer activity counts (ICC >0.94; 95% CI 0.91–0.97) in people post-stroke with no differences between workdays and weekend days.[114] In MS, test-retest ICC values were 0.91 and 0.88 for steps per day and activity counts per day (ActiGraph GT3X), respectively, over 6 months, although the ICC was smaller for people with greater disability (ICC = 0.672 for activity counts/day and ICC = 0.774 for steps/day).[37] In a direct comparison in MS, seven days of monitoring (ActiGraph 7164) produced an ICC of 0.93 whereas three days yielded an ICC of 0.80, with no difference noted between days of the week (weekdays or weekend days) when measuring walking activity or gait.[66] A 7-day period (using a TriTrac RT3 accelerometer) was most reliable in patients with stroke, MS or PD.[33] In PD, 24 hours of monitoring was found to be reliable to record a participants’ functional activity (average step count, inactive vs. active minutes using an activity monitor).[145] In spinocerebellar ataxia, internal consistency was highest with 7-days of monitoring, but 3 days of monitoring using a step activity monitor still correlated strongly with 7-day measures.[161]

Evidence of validity primarily comes from comparison of activity data collected remotely with established performance-based and self-report measures. In MS, number of steps per day correlates with the Expanded Disability Status Scale (EDSS), the Patient Determined Disease Steps (PDDS) scale, performance-based ambulatory measures in the clinic and patient-reported outcomes.[24, 31, 37, 38] Post-stroke, the ICC was high when comparing activity counts for the paretic and non-paretic hip (0.96), [114] but correlation was moderate when comparing activity with patient-reported activity questionnaires.[90] Post-stroke, activity counts for the upper extremity had high predictive value for good arm recovery; [98–100] both arms are used less than by healthy controls, and less arm activity correlates with increased impairment and reduced muscle activity measured by EMG.[98, 99, 103, 118–125] In TBI, activity counts were more accurate than questionnaires in characterizing levels of moderate to vigorous physical activity.[160] In spinocerebellar ataxia, average step count across 7 days correlated strongly with disability scores and moderately with walking speed.[161]

## Discussion

This systematic review examines a decade of literature on remote monitoring of physical activity in people with neurological diseases. Physical activity monitoring is feasible in these populations, including in those with impaired cognition. Some of the evidence was sparse: very few of the eligible studies used remote activity monitoring as an outcome for an intervention (9/134), [26, 30, 42, 68, 93, 104, 122, 123, 135] indicating that use of these tools in neurological populations is still primarily in an observational or validation phase. Nevertheless, the data in some diagnostic groups indicate that remote monitoring of physical activity can be a clinically useful way to assess activity status over time.

A wide array of variables can be used to measure physical activity. The most common are permutations of activity count or step count. However, other activity variables may provide better prognostic value in disease-specific situations. For example, length and number of moderate to vigorous activity bouts [86, 105] reflected differences better than total step count in some studies following stroke, [86, 116] whereas total step count, highest step rate in 1 minute, highest step rate in 5 minutes, and peak activity index appeared most reliable in others.[110] Detection of upper limb recovery via accelerometer measures of arm/upper extremity movement was also favored post-stroke, [98–100, 103, 104, 109, 112, 118–125] and may prove helpful in other populations, such as upper limb function in MS. In PD, average number of steps per day correlated with activity level and disease progression in many studies.[128, 132, 133] However, in a minority there was no correlation between activity count and patient-reported assessments of symptom severity.[140] Physical activity monitoring using specialized devices may also be used to predict fall-risk and measure missteps in PD, [129] functionality that, if replicated and validated, could be very be useful in other neurological populations, including MS and stroke.

Across diagnoses, physical activity is consistently lower in neurological populations than in those without neurological disease.[34–36, 83, 129, 148, 149] The total amount of activity or step counts measured via accelerometers is lower in MS (e.g.[63, 69]), dementia (e.g.[151, 153]), and stroke [118] than in controls. In people with moderate to severe PD, pattern of activity was different (sedentary bouts were longer) but total volume of sedentary time was similar to controls.[129] In those with mild to moderate PD, speed of turns was slower than in healthy controls, and reductions in daily ambulatory activity (volume of moderate to vigorous physical activity) were detected over a year, even without evident changes in clinical measures of gait or disease severity.[128]

Remote physical activity monitoring for durations of >24 hours was feasible in the neurological populations studied; [76] however, adherence was a potential concern. Post-stroke, the placement of sensors in pockets (confounding clothing movement with activity and increasing risk of leaving the device behind when changing clothes), impaired mental status, depression, and device discomfort (leading to withdrawal of 25% of patients from one study) all reduced adherence.[89, 91, 96] In PD, patients concerned with appearance also had reduced adherence (affecting over a quarter of participants in one study).[127] Physical activity monitoring for extended periods of time was well tolerated in people with Alzheimer's Disease, although adherence was lower (83%) compared to healthy controls (100%).[149] Tolerability was not recorded as a significant problem in studies involving people with MS, although adherence and loss of data from attrition was noted in several studies (S1a Table).

Intervention studies in stroke are heterogeneous with regards to adherence and walking performance. A circuit-based rehabilitation study aimed at increasing stroke patients’ amount and rate of walking in their home environment, found high adherence rates to the program.[108] Specific mention of device adherence was not recorded.[108] A separate intervention study recording steps per day during 4 weeks, reported ~25% attrition due to non-compliance.[106]

Interventional studies testing physical activity monitoring in stroke patients observed changes in clinical and patient-reported measures, but, perhaps in part due to inadequate adherence, failed to demonstrate changes in physical activity (average steps per day) in the home environment. [107, 108] Likewise, home intervention for increasing activity in people with PD observed improvements in strength, flexibility and a reduction in fear of falling, without noting changes is overall daily physical activity levels.[135] Studies in MS, however, indicated that Internet-based exercise interventions can help to increase physical activity (activity/ steps per day), and improve self-reported disease symptoms and self-efficacy over 6 months.[42, 68]

The few reviewed intervention studies using remote monitoring affirm that measuring activity levels of patients with minimal invasiveness in their natural environment has potential advantages over traditional self-reported and clinic-based measures. Self-reported measures are easy to obtain through questionnaires but are prone to recall bias. Performance-based measures in clinic can provide a useful snapshot of physical activity and may have prognostic value but are primarily measures of physical activity patients are *capable of* rather than how active patients *actually are* in their natural environment.[107, 108] Future intervention studies should continue measuring outcomes in multi-faceted ways as researchers gather more evidence of the relationship between the different categories of measures.

The accelerometer-based activity monitors used in many of the included studies are not primarily designed or marketed for consumer use, with current prices ranging from ~$200 to $600, which do not include software (~$2000) necessary for data analysis (S2 Table). Many commercially available monitors have not yet been evaluated in neurological populations. One recent study in healthy individuals showed no systematic bias when comparing step counts recorded via commercially available activity monitors (i.e. Fitbit) versus research grade accelerometers (ActiGraph).[162, 163] However, the accuracy of non-research grade activity monitors remains an active source of debate, [10, 19, 164–167] as does the failure of activity monitors to efficiently track many non-walking-based physical activities such as swimming, cycling, strength training and yoga.[168]

**Fig 2:**
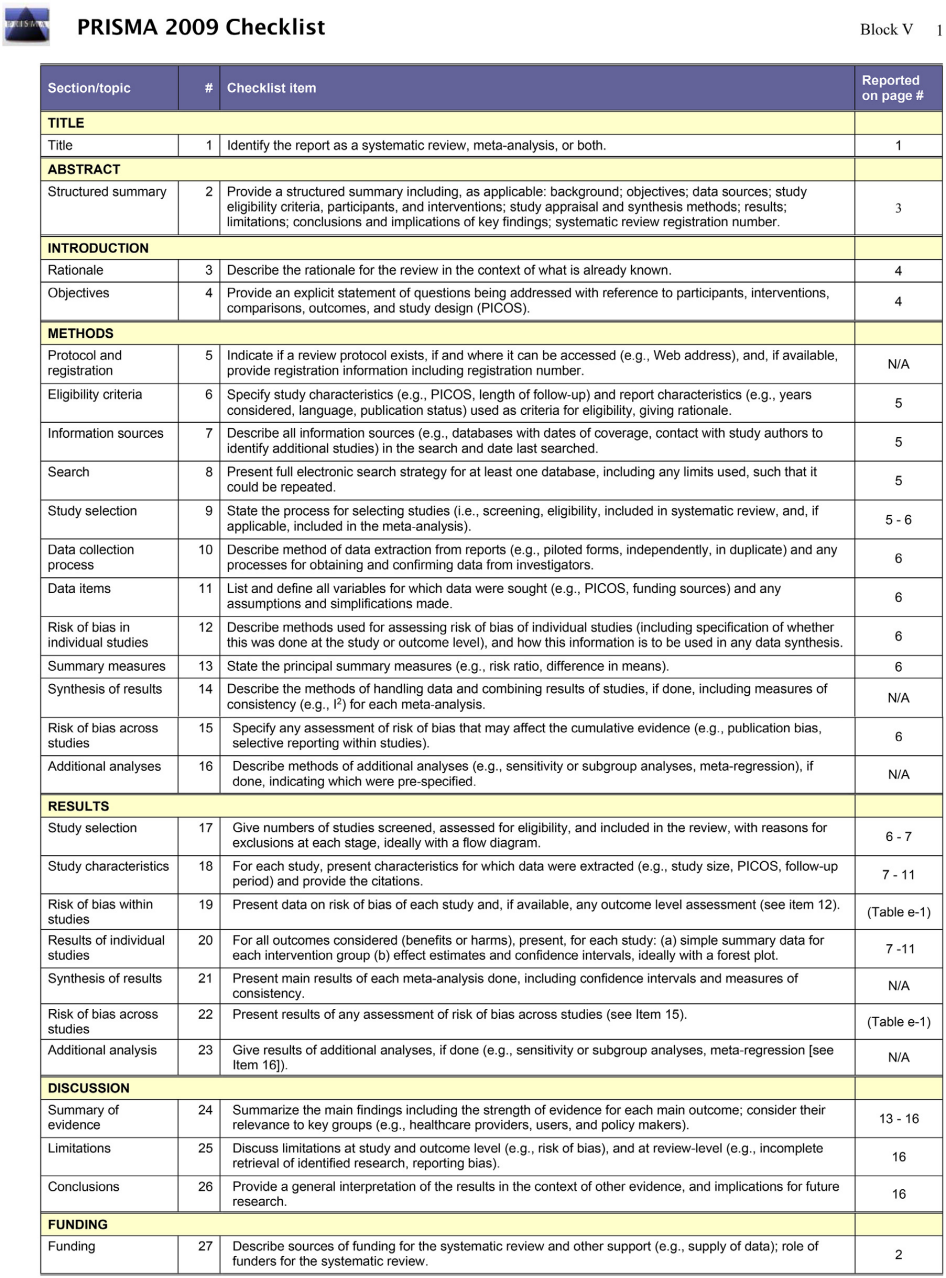
Checklist for Standardization of Reporting for Remote Physical Activity Monitoring in Neurological Disease. *From*: Moher D, Liberati A, Tetzlaff J, Altman DG, The PRISMA Group (2009). Preferred Reporting Items for Systematic Reviews and Meta-Analyses: The PRISMA Statement. PLoS Med 6(6): e1000097. doi:10.1371/journal.pmed1000097 For more information, visit: www.prisma-statement.org.

Lessons learned from this systematic review lead to several recommendations for translation of remote physical activity monitoring in neurological indications. 1) Remote physical activity monitoring research would benefit from standardization in reporting. We provide a checklist that might aid researchers and clinicians in future research and clinical use (Fig 2). 2) While remote monitoring devices and measurement protocols should be tested and validated in specific neurological conditions, solutions are likely to translate across neurological conditions that share patterns of functional impairment. 3) Activity monitors have the potential to be retooled with suites of variables specific to particular diagnostic indications. For example, a disease-specific remote monitoring suite for MS might include step and activity count, fall detection, upper extremity function and temperature sensors to correlate with possible heat-induced demyelination-related disability. Additional functionality could include reminders to exercise, take medication or keep to a schedule for bowel and bladder maintenance.[169] For all diagnostic groups, monitors could be tailored to track adherence to home exercise programs. If worn for longer periods of time, they could detect continuation of or changes in activity after specific punctate interventions (pharmacologic, medical, telehealth, or exercise-based) aimed to increase activity levels. Further studies are needed for longer periods of time (continuously for months/years) to determine the feasibility and responsiveness of activity monitoring devices for these purposes.

Limitations of this review include the focus on adults with neurological disease; lessons learned do not necessarily extend to pediatric populations with these conditions. This review also focuses specifically on physical activity monitoring and by necessity does not analyze advances in non-voluntary activities that can also be assessed via remote monitoring, such as seizure detection and sleep. Because only 9 of the 134 studies were interventional, our review does not include a meta-analysis.

In conclusion, this review records emerging evidence to support the use of remote physical activity monitoring in neurological care and neurorehabilitation. Because some patients already regularly perform such monitoring on themselves using commercial wearable devices or through their smartphones, providers also need to become familiar with these technologies and strategies for interpretation and to consider this knowledge translation when planning future studies.

## Supporting Information

S1 Fig PRISMA checklist. (TIFF)

S1 Table Risk of Bias for Individual Studies. (a = multiple sclerosis, b = stroke, c = Parkinson’s disease, d = Dementia/Alzheimer’s disease, and e = Multiple neurological disorders)
(DOCX)

S2 Table Summary of Common Monitors Used In Studies Monitoring Physical Activity for ≥ 24 Hours. (PDF)

S3 Table Level of Evidence Intervention studies. (DOCX)
