# Supplementary figures and images for: Remote Physical Activity Monitoring in Neurological Disease: A Systematic Review
Source: PLoS One. 2016 Apr 28;11(4):e0154335. doi: 10.1371/journal.pone.0154335 (PMC4849800; doi:10.1371/journal.pone.0154335)

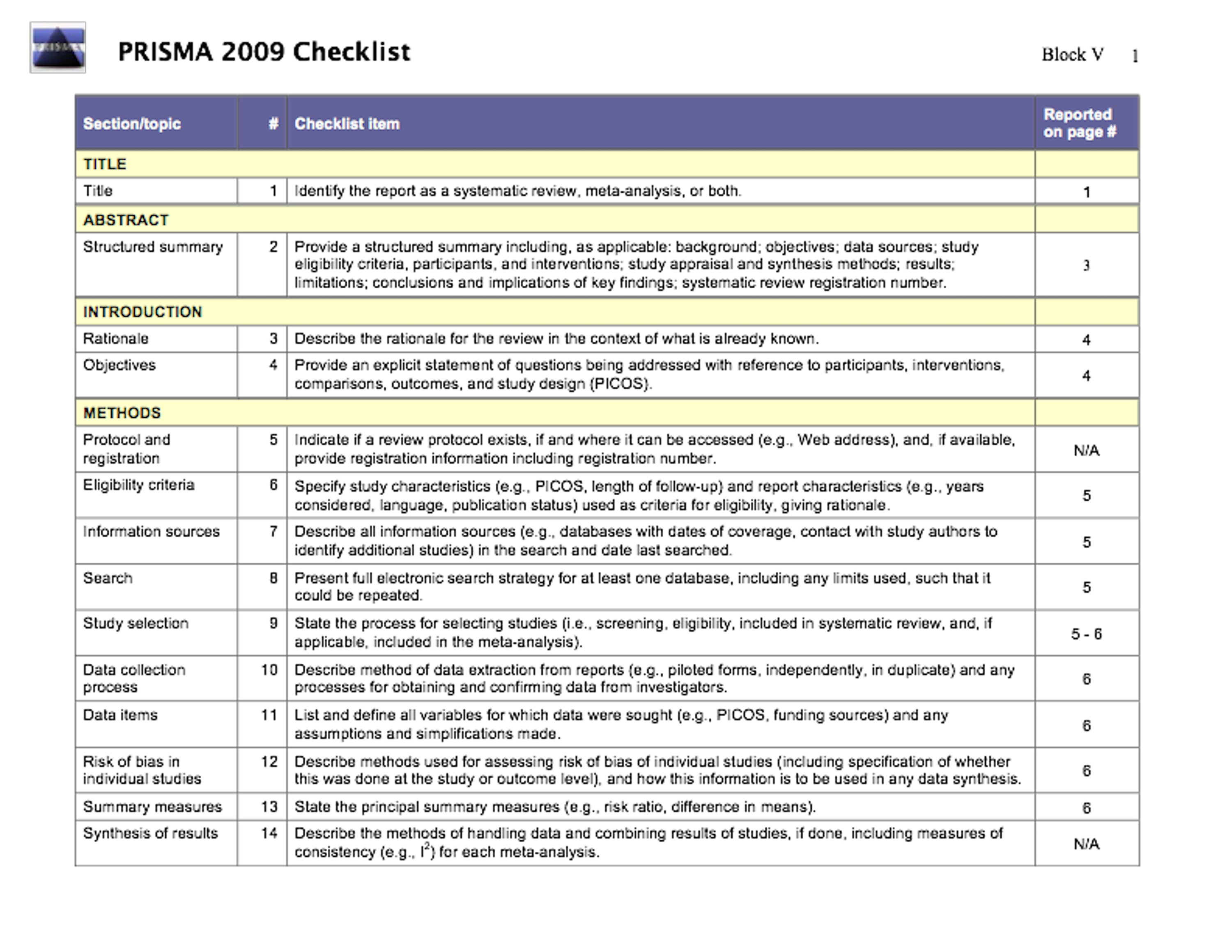

Supplement: S1 Fig — (TIFF) [file pone.0154335.s001.tiff]
