# Supplementary material for: Remote Physical Activity Monitoring in Neurological Disease: A Systematic Review
Source: PLoS One. 2016 Apr 28;11(4):e0154335. doi: 10.1371/journal.pone.0154335 (PMC4849800; doi:10.1371/journal.pone.0154335)
Supplement: S1 Table — (a = multiple sclerosis, b = stroke, c = Parkinson’s disease, d = Dementia/Alzheimer’s disease, and e = Multiple neurological disorders) (DOCX) [file pone.0154335.s002.docx]

| **eTable -1: Risk of Bias Table for Individual Studies** | | | | | | | | | |
| --- | --- | --- | --- | --- | --- | --- | --- | --- | --- |
| **Source** | **Design** | **Allocation Concealment** (Selection bias)* | **Random sequence generation** (Selection bias)* | **Blinding of Participants and Personnel** (Detection Bias) | **Blinding of Outcomes assessment** (Detection Bias) | **Incomplete outcome data** (Attrition Bias) | **Selective reporting** (Reporting Bias) | **Other: Diagnosis assessment** | |
| ***A: Multiple Sclerosis*** | |  |  |  |  |  |  | (**Definitive diagnosis of MS or self-reported**) | |
| Balantrapu et al, 2014 | Cross-Sectional |  |  | High | High** | Low | Low | Low | |
| Cavanaugh et al, 2011 | Cross-Sectional |  |  | High | High** | High | Low | Low | |
| Dlugonski et al, 2011 | Interventional † | High | Not RCT | High | High** | Low | Low | Low | |
| Dlugonski & Motl, 2012 | Cross-Sectional |  |  | High | High** | Unclear | Low | Low | |
| Dlugonski et al, 2013 | Cross-Sectional |  |  | High | High^ | Low | Low | High | |
| Doerksen et al, 2007 | Cross-Sectional |  |  | High | High^ | Low | Low | Low | |
| Filipovic Grcic et al, 2013 | Cross-Sectional |  |  | High | High** | Low | Low | Low | |
| Filipovic Grcic et al, 2011 | Interventional † | High | Not RCT | High | High** | Low | Low | Low | |
| Gijbels et al, 2010 | Cross-Sectional |  |  | High | High** | Low | Low | Low | |
| Gosney et al, 2007 | Cross-Sectional |  |  | High | High^ | Low | Low | Low | |
| Hale et al, 2008 | Longitudinal |  |  | High | High** | Low | Low | Low | |
| Klaren et al, 2013 | Mixed Observational (Cross-sectional & Longitudinal) |  |  | High | High** | Unclear | Unclear | Low |  |
| Klassen et al, 2008 | Cross-Sectional |  |  | High | High** | High*** | Low | Low |  |
| Kos et al, 2007 | Cross-Sectional |  |  | High | High** | High*** | Low | Low |  |
| Lamers et al, 2013 | Cross-Sectional |  |  | High | High** | Low | Low | Low |  |
| Learmonth et al, 2013 | Cross-Sectional |  |  | High | High** | Low | Low | Low |  |
| Learmonth et al, 2013 | Cross-Sectional |  |  | High | High** | Low | Low | Low |  |
|  |  |  |  |  |  |  |  |  |  |
| Morris et al, 2008 | Cross-Sectional |  |  | High | High** | Low | Unclear | High | |
| Motl et al, 2013a | Longitudinal |  |  | High | High** | High*** | Low | Low | |
| Motl et al, 2007b | Cross-Sectional |  |  | High | High** | Low | Unclear | Low | |
| Motl et al, 2010a | Cross-Sectional |  |  | High | High** | Low | Low | Low | |
| Motl & Dlugonski, 2011 | Interventional † | High | Not RCT | High | High^ ** | High*** | Low | Low | |
| Motl et al, 2011a | Mixed Observational (Cross-sectional & Longitudinal) |  |  | High | High** | Unclear | Unclear | Unclear | |
| Motl et al, 2011b | Cross-Sectional |  |  | High | High** | Unclear | Low | Low | |
| Motl et al, 2014b | Longitudinal |  |  | High | High** | Unclear | Low | Low | |
| Motl et al, 2012a | Cross-Sectional |  |  | High | High^ | Low | Unclear | Unclear | |
| Motl et al, 2014a | Longitudinal |  |  | High | High^** | Unclear | High | Unclear | |
| Motl et al, 2009a | Cross-Sectional |  |  | High | High** | Unclear | Low | Low | |
| Motl et al, 2006a | Cross-Sectional |  |  | High | High^** | Low | Low | Low | |
| Motl et al, 2007c | Cross-Sectional |  |  | High | High^** | Unclear | Low | Low | |
| Motl et al, 2008a | Cross-Sectional |  |  | High | High ** | Low | Low | Low | |
| Motl et al, 2010b | Cross-Sectional |  |  | High | High ** | Low | Low | Low | |
| Motl & McAuley, 2009a | Cross-Sectional |  |  | High | High ** | High*** | Low | Low | |
| Motl & McAuley, 2011 | Longitudinal |  |  | High | High ** | High*** | Low | Low | |
| Motl & McAuley, 2009b | Longitudinal |  |  | High | High ** | High*** | Low | Low | |
| Motl & McAuley, 2009c | Longitudinal |  |  | High | High ** | High*** | Low | Low | |
| Motl et al, 2013b | Cross-Sectional |  |  | High | High ** | High*** | Unclear | Low | |
| Motl et al, 2013c | Mixed Observational (Cross-sectional & Longitudinal) |  |  | High | High^ | Low | Unclear | High | |
|  |  |  |  |  |  |  |  |  | |
| Motl et al, 2012b | Cross-Sectional |  |  | High | High ** | Low | Unclear | Low | |
| Motl et al, 2009b | Cross-Sectional |  |  | High | High ** | Unclear | Low | Low | |
| Motl et al, 2006c | Cross-Sectional |  |  | High | High ** | Unclear | Unclear | High | |
| Motl et al, 2006b | Cross-Sectional |  |  | High | High ** | Unclear | Low | High | |
| Motl et al, 2007d | Cross-Sectional |  |  | High | High^ | Low | Low | Low | |
| Motl et al, 2008b | Cross-Sectional |  |  | High | High ** | Low | Low | Unclear | |
| Motl et al, 2007a | Cross-Sectional |  |  | High | High^ | High*** | Low | Low | |
| Pilutti et al, 2012 | Cross-Sectional |  |  | High | High ** | Low | Low | Low | |
| Pilutti et al, 2014 | RCT † | Unclear | Low | High | High^ ** | High*** | Low | Unclear | |
| Ranadive et al, 2012 | Cross-Sectional |  |  | High | High ** | Low | Low | Unclear | |
| Rietberg et al, 2014 | Cross-Sectional |  |  | High*** | High ** | High*** | Unclear | Low | |
| Rietberg et al, 2010 | Cross-Sectional |  |  | High | High ** | High*** | Unclear | Low | |
| Sandroff et al, 2013 | Longitudinal |  |  | High | High ** | High*** | High | Low | |
| Sandroff et al, 2012 | Cross-Sectional |  |  | High | High ** | Unclear | Low | Low | |
| Sandroff & Motl, 2013 | Cross-Sectional |  |  | High | High ** | Low | Low | Low | |
| Scott et al, 2011 | Cross-Sectional |  |  | High | High ** | Unclear | Unclear | Unclear | |
| Shammas et al, 2014 | Longitudinal |  |  | High | High ** | Low | Low | Low | |
| Snook et al, 2009 | Cross-Sectional |  |  | High | High ** | Unclear | Low | Low | |
| Snook & Motl, 2008 | Cross-Sectional |  |  | High | High ** | Low | High*** | High | |
| Sosnoff et al, 2010 | Cross-Sectional |  |  | High | High ** | Unclear | Low | Low | |
| Ward et al, 2013 | Cross-Sectional |  |  | High | High ** | Unclear | Low | Low | |
| Weikert et al, 2010 | Cross-Sectional |  |  | High | High ** | Unclear | Unclear | Low | |
| Weikert et al, 2012 | Cross-Sectional |  |  | High | High ** | Low | Low | High | |
| **Abbreviations and Key:** (Intervention studies highlighted)  RCT = Randomized control trial; Unclear= lack of information or uncertainty over the potential for bias  Source: http://handbook.cochrane.org/chapter_8/8_4_introduction_to_sources_of_bias_in_clinical_trials.htm  * Only examined for interventional studies / RCTs  ** Medium risk Detection bias (Participants blinded to results of accelerometers, not blinded to aim of monitoring or results of self-assessed physical activity measures. Assessors not blinded and or Personnel partially blinded)  *** Medium risk of Attrition bias (i.e. Missing data - taken into account in reporting results)  ^ ** Medium risk of detection bias. (i.e. Use of both pedometer - where the participant can see number of steps per day-- and accelerometer - where the participant can't see steps or data)  ^ Pedometer shows step count to participant - who then recorded this in a log every evening and reset the device; scope for human recording error and bias.  † Level of evidence for Interventional studies, all = level 2b or 2c: http://www.cebm.net/oxford-centre-evidence-based-medicine-levels-evidence-march-2009/ | | | | | | | | | |

| **Source** | **Design** | **Allocation Concealment** (Selection bias)* | **Random sequence generation** (Selection bias)* | **Blinding of participants and Personnel** (Detection Bias) | **Blinding of Outcomes assessment** (Detection Bias) | **Incomplete outcome data** (Attrition Bias) | **Selective reporting** (Reporting Bias) | **Other: Diagnosis assessment** |
| --- | --- | --- | --- | --- | --- | --- | --- | --- |
| ***B: Stroke*** |  |  |  |  |  |  |  | **Type of Stroke defined (low) undefined (high)** |
| Alzahrani et al, 2011 | Cross-sectional |  |  | High | High ** | Low | Low | High |
| Alzahrani et al, 2012 | Cross-sectional |  |  | High | High ** | Low | High | High |
| Alzahrani et al, 2009 | Cross-sectional |  |  | High | High ** | Low | High | High |
| Askim et al, 2013 | Cross-sectional |  |  | High *** | High ** | High*** | High | Low |
| Baert et al, 2012 | Cross-sectional |  |  | High | High^ | Low | Unclear | Low |
| Barak et al, 2014 | Cross-sectional |  |  | High *** | High ** | High*** | Low | Low |
| Bowden et al, 2008 | Cross-sectional |  |  | High | High ** | Low | Low | High |
| Butler & Evenson, 2014 | Cross-sectional |  |  | High | High ** | High*** | High | High |
| Danks et al, 2014 | Interventional † | High | Not RCT | High | High ** | High*** | Unclear | High |
| De Niet et al, 2007 | Cross-sectional |  |  | High *** | High ** | Low | High | Low |
| Dobkin et al, 2011 | Cross-sectional |  |  | High | High ** | High*** | Low | High |
| Frazer et al, 2013 | Cross-sectional |  |  | High | High ** | High*** | Low | High |
| Fulk et al, 2010 | Cross-sectional |  |  | High | High ** | Low | Low | High |
| Fulk et al, 2014 | Cross-sectional |  |  | High | High^ ** | Low | Unclear | (N/A) TBI / Stroke |
| Gebruers et al, 2014 | Cross-sectional |  |  | High*** | High ** | High*** | Low | Low |
| Gebruers et al, 2013 | Cross-sectional |  |  | High*** | High ** | High*** | Low | Low |
| Gebruers et al, 2008 | Cross-sectional |  |  | High | High ** | High*** | Low | Low |
| Haeuber et al, 2004 | Cross-sectional |  |  | High | High ** | Low | Low | Low |
| Knarr et al, 2013 | Cross-sectional |  |  | High | High ** | High | Low | High |
| Lang et al, 2007 | Cross-sectional |  |  | High | High ** | Low | Low | Low |
| Lemmens et al, 2014 | RCT † | High | Medium/  low | High | High ** | Low | Low | High |
| Manns & Baldwin, 2009 | Cross-sectional |  |  | High | High ** | Low | High | High |
| Michielsen et al, 2012 | Cross-sectional |  |  | High *** | High ** | Low | Low | High |
| Mudge & Stott, 2009 | Cross-sectional |  |  | High | High ** | Low | High | High |
| Mudge & Stott, 2008 | Cross-sectional |  |  | High | High ** | High | Low | High |
| Rand & Eng, 2012 | Cross-sectional |  |  | High | High ** | High | Low | Low |
| Rand et al, 2010 | Cross-sectional |  |  | High | High ** | High | Low | High |
| Rand et al, 2009 | Cross-sectional |  |  | High | High ** | Low | Low | Low |
| Reiterer et al, 2008 | Longitudinal |  |  | High | High ** | High*** | Unclear | Low |
| Robinson et al, 2011 | Cross-sectional |  |  | High | High ** | High*** | Low | High |
| Roos et al, 2012 | Cross-sectional |  |  | High | High ** | High*** | Low | High |
| Seitz et al, 2011 | Cross-sectional |  |  | High | High ** | Low | Low | Low |
| Shim et al, 2014 | Cross-sectional |  |  | High | High ** | Low | Unclear | High |
| Strommen et al, 2014 | Cross-sectional |  |  | High | High ** | High*** | Low | Low |
| Thrane et al, 2011 | Cross-sectional |  |  | High | High ** | High*** | Low | Low |
| Uswatte et al, 2005 | Interventional † | High | Medium/  low | High | High ** | Low | Unclear | High |
| Uswatte et al, 2006 | Interventional † | High | Medium/  low | High | High ** | High*** | Low | Low |
| Uswatte et al, 2009 | Cross-sectional |  |  | High | High ** | Unclear | Unclear | High |
| Van der Pas et al, 2011 | Cross-sectional |  |  | High | High ** | Low | Low | High |
| **Abbreviations and Key:** (Intervention studies highlighted)  RCT = Randomized control trial; N/A = not applicable; Unclear = lack of information or uncertainty over the potential for bias; Medium = simple randomization (medium/low = incorporates method to reduce bias of simple randomization to groups)  Source: http://handbook.cochrane.org/chapter_8/8_4_introduction_to_sources_of_bias_in_clinical_trials.htm  * Only for interventional / RCT  ** Medium risk Detection bias (Participants blinded to results of accelerometers, not blinded to aim of monitoring or results of self-assessed physical activity measures. Assessors not blinded and or Personnel partially blinded)  *** Medium risk of Attrition bias (i.e. Missing data - taken into account in reporting results)  ^ ** Medium risk of detection bias. (i.e. Use of both pedometer - where the participant can see number of steps per day-- and accelerometer - where the participant can't see steps or data)  ^ Pedometer shows step count to participant - who then recorded this in a log every evening and reset the device; scope for human recording error and bias  † Level of evidence for Interventional studies, all = level 2b or 2c: http://www.cebm.net/oxford-centre-evidence-based-medicine-levels-evidence-march-2009/ | | | | | | | | |

| **Source** | **Design** | **Allocation Concealment** (Selection bias)* | **Random sequence generation** (Selection bias)* | **Blinding of participants and Personnel** (Detection Bias) | **Blinding of Outcomes assessment** (Detection Bias) | **Incomplete outcome data** (Attrition Bias) | **Selective reporting** (Reporting Bias) | **Other: Diagnosis assessment** |
| --- | --- | --- | --- | --- | --- | --- | --- | --- |
| ***C: Parkinson’s Disease*** | |  |  |  |  |  |  | **Definitive diagnosis of PD** |
| Cancela et al, 2014 | Cross-sectional |  |  | High | High ** | Unclear | Low | Low |
| Cavanaugh et al, 2012 | Longitudinal |  |  | High | High ** | High*** | Low | Low |
| Chastin et al, 2010 | Cross-sectional |  |  | High | High ** | Unclear | Unclear | Low |
| Dontje et al, 2013 | Cross-sectional |  |  | High | High ** | Low | Low | Low |
| El-Gohary et al, 2013 | Cross-sectional |  |  | High | High ** | Unclear | Low | Unclear |
| Ellis et al, 2011 | Cross-sectional |  |  | High | High ** | Low | Low | Low |
| Ford et al, 2010 | Cross-sectional |  |  | High | High ** | Unclear | Low | Low |
| Garcia Ruiz & Sanchez Bernardos, 2008 | Cross-sectional |  |  | High | High ** | Unclear | Unclear | Low |
| Hideyuki & Hitoshi, 2011 | Cross-sectional |  |  | High | High ** | High | Low | Unclear |
| Hideyuki & Hitoshi, 2014 | Interventional † | High | Not RCT | High | High ** | High | Low | Low |
| Iluz et al, 2014 | Cross-sectional |  |  | High | High ** | Low | Unclear | Low |
| Lord et al, 2013 | Cross-sectional |  |  | High | High ** | High | High | Low |
| Moore et al, 2011 | Cross-sectional |  |  | High** | High ** | Low | Low | Unclear |
| Pan et al, 2007 | Cross-sectional |  |  | High | High ** | Unclear | Low | Low |
| Rochester et al, 2006 | Cross-sectional |  |  | High | High ** | High | Low | Low |
| Wallen et al, 2014a | Cross-sectional |  |  | High | High^ ** | High | Low | Low |
| Wallen et al, 2014b | Cross-sectional |  |  | High | High ** | Unclear | Low | Low |
| Weiss et al, 2014 | Cross-sectional |  |  | High | High ** | High | Low | Low |
| White et al, 2007. | Longitudinal |  |  | High** | High ** | Unclear | Low | Low |
| Yoneyama et al, 2013 | Cross-sectional |  |  | High | High ** | Low (N/A) | High (N/A) | Unclear |
| Busse et al, 2004”” | Longitudinal |  |  | High | High ** | Low | Unclear | Unclear (PD, MS, Neuromuscular) |
| **Abbreviations and Key:** (Intervention studies highlighted)  RCT = Randomized control trial, PD = Parkinson’s disease, MS = Multiple sclerosis, N/A = not applicable, Unclear: lack of information or uncertainty over the potential for bias  Source: http://handbook.cochrane.org/chapter_8/8_4_introduction_to_sources_of_bias_in_clinical_trials.htm  * Only for interventional / RCT  ** Medium risk Detection bias (Participants blinded to results of accelerometers, not blinded to aim of monitoring or results of self-assessed physical activity measures. Assessors not blinded and or Personnel partially blinded)  *** Medium risk of Attrition bias (i.e. Missing data - taken into account in reporting results)  ^ ** Medium risk of detection bias. (i.e. Use of both Pedometer - where the participant can see number of steps per day. Accelerometer - where the participant can't see steps or data)  ^ Pedometer shows step count to participant - who then recorded this in a log every evening and reset the device. Scope for human recording error and bias  “” Multiple neurological conditions included in the study  † Level of evidence for Interventional studies, all = level 2b or 2c: http://www.cebm.net/oxford-centre-evidence-based-medicine-levels-evidence-march-2009/ | | | | | | | | |

| **Source** | **Design** | **Allocation Concealment** (Selection bias)* | **Random sequence generation** (Selection bias)* | **Blinding of participants and Personnel** (Detection Bias) | **Blinding of Outcomes assessment** (Detection Bias) | **Incomplete outcome data** (Attrition Bias) | **Selective reporting** (Reporting Bias) | **Other: Diagnosis assessment** |
| --- | --- | --- | --- | --- | --- | --- | --- | --- |
| ***D: Alzheimer’s Disease*** | |  |  |  |  |  |  | **Neuro/Psy Diagnosis defined:** |
| David et al, 2012 | Cross-sectional |  |  | High | High ** | High | Low | Low |
| Erickson et al, 2013 | Cross-sectional |  |  | High | High ** | High | Unclear | Low |
| Gietzelt et al, 2014 | Longitudinal |  |  | High | High ** | High | Low | Low |
| Gietzelt et al, 2013 | Cross-sectional |  |  | High | High ** | Low | Low | Unclear |
| Greiner et al, 2007 | Cross-sectional |  |  | High | High ** | Unclear | High | Low |
| Hoffmeyer et al, 2012 | Cross-sectional |  |  | High | High ** | Low | Low | Low |
| James et al, 2012 | Cross-sectional |  |  | High | High ** | Unclear | High | Unclear |
| Kirste et al, 2014 | Cross-sectional |  |  | High | High ** | High | Low | Low |
| Nagels et al, 2007 | Cross-sectional |  |  | High | High ** | High | Unclear | Low |
| Nagels et al, 2006 | Cross-sectional |  |  | High | High ** | High | Low | Low |
| Yuki et al, 2012 | Longitudinal |  |  | High | High ** | High | Unclear | Low |
| ***E: Traumatic Brain Injury / Ataxia / Multiple Conditions*** | | | |  |  |  |  |  |
| Hassett et al, 2014 | Cross-sectional |  |  | High | High ** | Low | Low | Low (TBI) |
| Subramony et al, 2012 | Cross-sectional |  |  | High | High ** | Low | Low | Low (Ataxia) |
| Fulk et al, 2014”” | Cross-sectional |  |  | High | High^ ** | Low | Unclear | Unclear (TBI / Stroke) |
| Hale et al, 2008”” | Longitudinal |  |  | High | High** | Low | Low | Low (MS, PD, Stroke) |
| Busse et al, 2004”” | Longitudinal |  |  | High | High ** | Low | Unclear | Unclear (PD, MS, Neuromuscular) |
| **Abbreviations and Key:** RCT = Randomized control trial, TBI = traumatic brain injury, PD = Parkinson’s disease, MS = Multiple sclerosis, N/A = not applicable, Neuro/Psy = Neurological or Psychological, Unclear: lack of information or uncertainty over the potential for bias  * Only for interventional / RCT  ** Medium risk Detection bias (Participants blinded to results of accelerometers, not blinded to aim of monitoring or results of self-assessed physical activity measures. Assessors not blinded and or Personnel partially blinded)  ^ ** Medium risk of detection bias (i.e. Use of both Pedometer - where the participant can see number of steps per day. Accelerometer - where the participant can't see steps or data)  “” Multiple conditions included in the study  Source: <http://handbook.cochrane.org/chapter_8/8_4_introduction_to_sources_of_bias_in_clinical_trials.htm> | | | | | | | | |
