# Supplementary material for: Remote Physical Activity Monitoring in Neurological Disease: A Systematic Review
Source: PLoS One. 2016 Apr 28;11(4):e0154335. doi: 10.1371/journal.pone.0154335 (PMC4849800; doi:10.1371/journal.pone.0154335)
Supplement: S2 Table — (PDF) [file pone.0154335.s003.pdf]

Table e-2: Summary Of Common Monitors Used In Studies Monitoring Physical Activity For ≥ 24 Hours.

Name	Manufacturer	Acceleration directions detected	Device Dimensions (cm)	Placement	Average Cost*	Main Outcomes measured	Comments
**ActiGraph Model 7164 (a)λ**	ActiGraph LLC Pensacola, FL	1 (uniaxial) **	5.1 x 4.1 x 1.5 (45.5g)	Hip, wrist or ankle	$225-299<br>Software $1495	Activity count, step count, EE, activity intensity level	> Placement on R or non-dom./unaffected hip
**ActiGraph Model GT3X (a)λ**	ActiGraph LLC Pensacola, FL	3 (triaxial) **	4.6 x 3.3 x 1.5 (19g)	Hip, wrist or ankle	$225-299<br>Software $1495	Activity count, step count, EE, activity intensity level	> Placement on R or non-dom./unaffected hip
**StepWatch Activity Monitor (SAM)**	Modus Health Inc, Washington DC	Microprocessor-controlled activity monitor	6.5 x 5.0 x 1.5 (65g)	Ankle	$525<br>Software	Step count, active vs inactive minutes, step gait characteristics	Greater accuracy for slow walking speeds.

**(b)λ**					$1470		
**StepWatch 3 Activity Monitor**	Orthocare Innovations, Oklahoma City, Oklahoma	Microprocessor-controlled activity monitor	6.5 x 5.0 x 1.5 (65g)	Ankle	$525<br>Software $1470	Step count, active vs inactive minutes, step gait characteristics	Greater accuracy for slow walking speeds.
**Yamax SW-200 digiwalker Step Pedometer (c)**	Yamax-Digiwalker, Warminster, PA	Pedometer – spring loaded leaver arm	5.0 x 3.8 x 1.4 (21g)	Clip to multiple locations	$13 -19.50	Step count	___
**TriTrac RT3 (d)**	StayHealthy Inc., Monrovia, CA, USA	3 (triaxial) **	10.8 x 6.8 x 3.3 (170.4g)	Waist	~$500	Net vector magnitude counts, EE, step count	> Placement on posterior waist
**DynaPort Hybrid (e)**	McRoberts, The Hague,	3 (triaxial) Accel – 3 (triaxial) gyroscope.	87 x 45 x 14 (74g)	Lower back (level	Hardware EUR 695	Physical activity (steps, intensity & postures), EE,	___

	Netherlands			of L4-5)	Module – EUR 200^	sleep	
**Shimmer3 IMU (f)**	Shimmer Boston MA, USA	Accelerometer, gyroscope & magnetometer	51 x 34 x 14 (23.6g)	Multiple locations	~$558		> worn on the wrist
**Intelligent Device for Energy Expenditure and Activity (g)**	MiniSun Company, 935 E. MillCreek Dr., Fresno, California, 93720, USA	1 recorder and 5 sets of sensors	1cm^2(5 sensors) (58g)	Multiple locations		Activity counts (walking, stairs, transitions), Time spent: on feet vs not on feet.	

Abbreviations: EE = energy expenditure, > = mostly, R = right, L = left, non-dom = non-dominant, Accel = Accelerometer.

λ Approved by the Food and Drug Administration (FDA) (StepWatch = marketing clearance as a class II device)

* Cost data was obtained from the manufacturer website as well as major retail sites (www.amazon.com when relevant), date accessed 06.14.2015.

** Using a piezoelectric bender element or solid-state accelerometer that produces an electric signal proportionate to the force acting on it during movement.

^ 1 module = EUR 200, 2 modules = EUR 300, 3 modules = EUR 375 and 4 modules = EUR 400

Websites: a) http://www.actigraphcorp.com, b) https://modushealth.com, (c) http://www.yamax-digiwalker.com, (d) https://www.stayhealthy.com, (e) https://www.mcroberts.nl/, (f) http://www.shimmersensing.com/shop/consensys-base6-imu-development-kits

NB: Common = used in ≥ 2 studies
