# Supplementary material for: Remote Physical Activity Monitoring in Neurological Disease: A Systematic Review
Source: PLoS One. 2016 Apr 28;11(4):e0154335. doi: 10.1371/journal.pone.0154335 (PMC4849800; doi:10.1371/journal.pone.0154335)
Supplement: S3 Table — (DOCX) [file pone.0154335.s004.docx]

| Table e-3. Level of Evidence for Intervention Studies | | | | |
| --- | --- | --- | --- | --- |
| Source | **Design** | **Level of Evidence (1a -5) *** | | |
|  |  |  |  |  |
| *Multiple Sclerosis* | |  |  |  |
| Dlugonski et al, 2011 | Intervention | 2c |  |  |
| Filipovic Grcic et al, 2011 | Intervention | 2c |  |  |
| Motl & Dlugonski, 2011 | Intervention | 2c |  |  |
| Pilutti et al, 2014 | RCT | 2b |  |  |
| *Stroke* | |  |  |  |
| Danks et al, 2014 | Intervention | 2b |  |  |
| Lemmens et al, 2014 | RCT | 2b |  |  |
| Michael et al, 2009 | Intervention | 2c |  |  |
| Mudge et al, 2009 | Intervention | 2b |  |  |
| Moore et al, 2010 | Randomized crossover Trial | 2b |  |  |
| Uswatte et al, 2005 | Intervention | 2c |  |  |
| Uswatte et al, 2006 | Intervention | 2b |  |  |
| *Parkinson’s Disease* | |  |  |  |
| Hideyuki & Hitoshi, 2014 | Intervention | 2c |  |  |
| **Abbreviations and Key:**  MS = multiple sclerosis, PD = Parkinson’s disease, RCT = randomized control trial  Source*: http://www.cebm.net/oxford-centre-evidence-based-medicine-levels-evidence-march-2009/ | | | |  |
